# A 11B-NMR Method for the In Situ Monitoring of the Formation of Dynamic Covalent Boronate Esters in Dendrimers

**DOI:** 10.3390/polym16233258

**Published:** 2024-11-23

**Authors:** Yi-Wen Yao, Ching-Hua Tsai, Chih-Yi Liu, Fang-Yu Wang, Sodio C. N. Hsu, Chun-Cheng Lin, Hui-Ting Chen, Chai-Lin Kao

**Affiliations:** 1 Department of Medicinal and Applied Chemistry, Kaohsiung Medical University, Kaohsiung 807, Taiwancrime93@yahoo.com.tw (C.-H.T.); u109021119@gap.kmu.edu.tw (C.-Y.L.); u109021115@gap.kmu.edu.tw (F.-Y.W.); sodiohsu@kmu.edu.tw (S.C.N.H.); cclin66@mx.nthu.edu.tw (C.-C.L.); 2 Department of Chemistry, National Tsing Hua University, Hsinchu 300, Taiwan; 3 Department of Pharmacy, National Yang Ming Chiao Tung University, Taipei 112, Taiwan; 4 Department of Medical Research, Kaohsiung Medical University Hospital, Kaohsiung 807, Taiwan; 5 Drug Development and Value Creation Research Center, Kaohsiung Medical University, Kaohsiung 807, Taiwan; 6 Center for Tropical Medicine and Infectious Disease Research, Kaohsiung Medical University, Kaohsiung 807, Taiwan; 7 Department of Chemistry, National Sun Yat-sen University, Kaohsiung 804, Taiwan; 8 College of Professional Studies, National Pingtung University of Science and Technology, Pingtung 912, Taiwan

**Keywords:** dynamic covalent chemistry, ^11^B NMR, boronate ester, amine-coordinated boronate ester

## Abstract

The in situ monitoring of dynamic covalent macromolecular boronate esters represents a difficult task. In this report, we present an in situ method using fluoride coordination and ^11^B NMR spectroscopy to determine the amount of boronate esters in a mixture of boronic acids and cis-diols. With fluoride coordination, the boronic acid and boronate esters afforded trifluoroborate and fluoroboronate esters, giving identical resonances at 3 and 9 ppm in the ^11^B NMR spectra. The same titration did not alter the resonance of amine-coordinated boronate esters, which gave resonances of 14 ppm in the ^11^B NMR spectra. Therefore, boronic acids, boronate esters, and amine-coordinated boronate esters gave three identical resonances, and the ratio of each component was obtained by deconvolution for a further equilibrium analysis. This method monitored the conversion among three species in various conditions, including separation. Accordingly, boronate esters were more stable after precipitation than chromatography, in which 29% and 20% of boronate esters were lost after purification. This method was applied to study the reaction between the boronic acid-decorated defect lysine dendron (**16**) and dopamine. No boronic acid signal was observed after adding 1 equivalent of dopamine; no boronic acid signal was observed in the NMR spectrum. According to the spectrum, the product contains 65% boronate ester and 35% N–B-coordinated derivatives. This method helps identify the presence of the three intermediates and provides more insights into this reaction.

## 1. Introduction

The reversible binding of boronic acids and *cis*-diols [1,2] to boronate esters offers an unprecedented toolbox for polymer science [3,4,5,6,7]. Boronate esters provide a reversible and adaptive polymer framework, which has been developed into smart materials such as shape memory polymers [8], self-healing materials [9,10], hydrogels [11,12], and responsive materials [13,14]. The formation of boronate esters depends on the in situ ratio and concentrations of boronic acids and diols. Therefore, the ratio of boronate esters in a mixture of boronic acids and *cis*-diols is difficult to determine. Recently, boronate ester polymers were developed as delivery vesicles to carry proteins [15], carbohydrates [10], and drugs as therapeutic agents [16,17]. For clinical applications, it is essential to determine the ratio of boronate esters in the mixture of boronic acids and cis-diols for predicting efficacy and estimating toxicity, as the number of therapeutic agents is significant.

A molecule functionalized with boronic acid and a fluorescence moiety emits different wavelengths after forming a boronate ester [18]. However, this strategy requires a sophisticated structural design and tedious synthesis, which limits its application. In addition, boronate esters and boronic acids give different signals in Fourier-transform infrared (FTIR) spectra [19]. However, the signals of boronic acids (900–1000 cm^−1^) are in the fingerprint region, which overlaps with typical signals of organic functional groups. This drawback limits the scope of the FTIR method in the determination of boronate esters. It was reported that surface-enhanced Raman spectroscopy (SERS) can be used to monitor boronate ester formation [20]. However, SERS requires a functional group for metal surface attachment and an extra moiety for each analyte. These requirements make the preparation of materials and the interpretation of results more challenging. The above spectroscopic methods need considerations of either arranging molecular structures or implanting specific dyes for spectroscopy. All of these requirements raise barriers to monitoring boronate formation. Additionally, research on macromolecules is more challenging because of their large number and proximity of boronic acids and boronate esters [21]. Therefore, because of these limitations, a new convenient method is necessary for developing in situ boronic acid-containing macromolecules.

**Figure 1:**
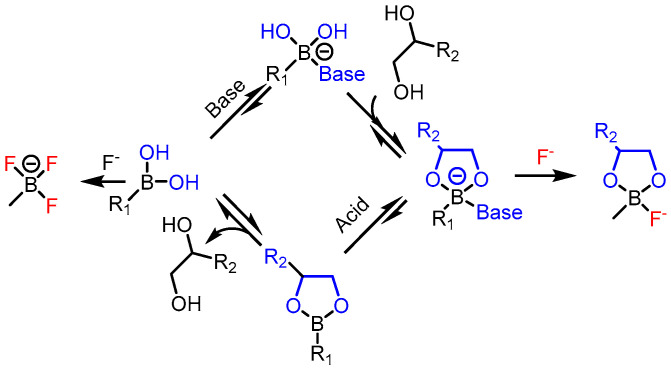
General reaction of boronic acids.

Boronic acid consists of sp^2^ hybridization with one empty *p*-orbital on the boron center, which allows for nucleophile coordination [22,23,24] and results in a tetracoordinated boron center with sp^3^ hybridization [22]. Remarkably, the tetrahedral boronate anion exhibits a preference for forming boronate esters rather than the trigonal boronic acid form [23,24] (Figure 1). Therefore, the hybridization of the boron atoms of boronic acids and boronate esters can generate different resonances in ^11^B nuclear magnetic resonance (^11^B NMR) spectra. Meanwhile, fluorides coordinate boronic acids and boronate esters to mono- and tri-fluorinated boronates, respectively [25,26,27,28,29]. Alongside strong electronegativity, two more fluorides on boronate acids create different environments from corresponding fluoride-coordinated boronate esters. This leads to different resonances of these two derivatives in ^11^B NMR spectra. Accordingly, an ^11^B NMR analysis is a potential method for the in situ measuring of the ratios of each molecule in a mixture and monitoring the interconversion between boronic acids and boronate esters.

## 2. Materials and Methods

### 2.1. Materials and Chemicals

A G2 poly(amidoamine) (PAMAM) dendrimer was purchased from Dendritech. 4-carboxyphenylboronic acid (CPBA, 98%, AK sci., Union City, CA, USA); dopamine hydrochloride (99%, Sigma Aldrich, St. Louis, MO, USA); benzotriazol-1-yloxytripyrrolidinophosphonium hexafluorophosphate (PyBOP, ≥99.0%, Sigma Aldrich, St. Louis, MO, USA); *N*-hydroxysuccinimide (≥99.0%, Sigma Aldrich, St. Louis, MO, USA); 1-ethyl-3-(3-dimethylaminopropyl)carbodiimide hydrochloride (EDC·HCl, 98%, Sigma Aldrich, St. Louis, MO, USA); *N*,*N*,*N*′,*N*′-tetramethyl-*O*-(1*H*-benzotriazol-1-yl)uronium hexafluorophosphate (HBTU, ≥98.0%, Sigma Aldrich, St. Louis, MO, USA); 1-hydroxybenzotriazole (HOBt, ≥97.0%, Sigma Aldrich, St. Louis, MO, USA); tetra-*n*-butylammonium fluoride (TBAF, 0.903 g/mL, 1 M soln. in THF, Sigma Aldrich, St. Louis, MO, USA); β-alanine (99%, Boc-Lys(Boc)-OH ≥98%, Sigma Aldrich, St. Louis, MO, USA); phenylalanine (≥97.0%, Sigma Aldrich, St. Louis, MO, USA); *N*^α^,*N*^ε^-di-Fmoc-L-lysine (Fmoc-Lys(Fmoc)-OH, ≥98%, Sigma Aldrich, St. Louis, MO, USA); *N*α-*t*-Boc-*N*ε-Fmoc-L-lysine (Boc-Lys(Fmoc)-OH, ≥98%, Sigma Aldrich, St. Louis, MO, USA); rink amide resin (loading ratio 0.3 mmol/g, 100–200 mesh, Sigma Aldrich, St. Louis, MO, USA); di-tert-butyl dicarbonate (0.95 g/mL, ≥98.0%, Sigma Aldrich, St. Louis, MO, USA); hydrochloric acid (HCl, 37%, Sigma Aldrich, St. Louis, MO, USA); thionyl chloride (SOCl_2_, 1.631 g/mL, ≥97.0%, Sigma Aldrich, St. Louis, MO, USA); sodium hydroxide (NaOH, ≥97.0%, Sigma Aldrich, St. Louis, MO, USA); triethylamine (Et_3_N, ≥99.5%, Sigma Aldrich, St. Louis, MO, USA); *n*-methyl morpholine (NMM, 0.92 g/mL, ≥99.5%, Sigma Aldrich, St. Louis, MO, USA); and piperidine (0.862 g/mL, ≥99%, Sigma Aldrich, St. Louis, MO, USA) were used in this study. The chemicals were used without further purification. Analytical thin-layer chromatography (TLC, Merck, Rahway, NJ, USA) was performed using silica gel 60 F254 plates that were 0.2 mm thick with a UV light (254 and 364 nm) as a revelator. Chromatography was prepared on silica gel (200–300 mesh, Merck, Rahway, NJ, USA). Size exclusion chromatography was prepared on Sephadex® LH-20 (25–100 µM, Sigma Aldrich, St. Louis, MO, USA). D-solvents were purchased from Merck Co. (Rahway, NJ, USA). NMR spectra were obtained from a JEOL 400 MHz spectrometer (Tokyo, Japan). Deconvolution was processed by the software Delta (version 5.0.5) with the Lorentzian deconvolution method. ESI mass spectra were recorded using the FT–ESI–MS system (Bruker Solarix, Billerica, MA, USA). MALDI mass spectra were recorded using the MALDI–TOF–MS system (BRUKER ultraflextreme, Billerica, MA, USA).

### 2.2. Synthesis of 4-Carboxypheny Boronate Ester Dopamine (***2***) (Scheme 1)

A solution of 4-carboxypheny boronic acid (50.0 mg, 0.30 mmol) in toluene (2.0 mL) and EtOH (1.0 mL) was added dropwise to a solution of dopamine hydrochloride (57.1 mg, 0.30 mmol) in toluene (3.0 mL) at an ambient temperature. The reaction mixture was then heated to reflux for 10 d with a Dean–Stark apparatus. The mixture was filtered and washed by toluene (1.0 mL) to derive the desired compound as a white solid (82.7 mg, yield: 97%). ^1^H NMR (DMSO-*d*_6_, 400 MHz): δ 7.87–7.80 (m, 4H); 6.64 (d, 1H, *J* = 8.1 Hz); 6.58 (d, 1H, *J* = 2.1 Hz); 6.45 (dd, 1H, *J* = 8.0, 2.1 Hz); 2.87 (t, 2H, *J* = 8.0 Hz); 2.64 (t, 2H, *J* = 8.0 Hz). ^13^C NMR (DMSO-*d*_6_, 100 MHz): δ 167.99, 145.83, 144.59, 139.86, 134.63, 132.46, 131.91, 128.61, 128.45, 119.75, 116.58, 116.29, 32.90. ^11^B NMR (DMSO-*d*_6_, 128 MHz): δ 27.32.

**Scheme 1:**
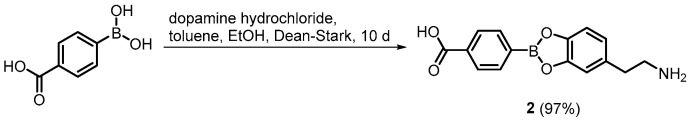
Synthetic scheme of 4-Carboxypheny boronate ester dopamine (**2**).

### 2.3. Synthesis of (G:2)-Dendri-PAMAM-(CPBA)_15_ (***6***)

Benzotriazol-1-yloxytripyrrolidinophosphonium hexafluorophosphate (PyBOP) (511.4 mg, 0.98 mmol) and Et_3_N (93.7 mg, 0.13 mL, 0.93 mmol) were added to a solution of 4-carboxyphenylboronic acid (163.1 mg, 0.98 mmol) in anhydrous DMF (10.0 mL) and stirred at an ambient temperature for 40 min. The resulting mixture was slowly added to a solution of a G2 PAMAM dendrimer (100.0 mg, 30.7 mmol) in anhydrous DMF (10.0 mL) and EtOH (1.0 mL) at an ambient temperature. After 4 d of stirring at 45 °C, the mixture was filtered and washed by DMF (2.0 mL). The crude product was purified by chromatography Sephadex^®^ LH-20 (Sigma Aldrich, St. Louis, MO, USA) to derive the desired compound as a colorless solid (85.7 mg, yield: 51%). ^1^H NMR (CDCl_3_, 400 MHz): δ 7.73 (s, 54H); 3.56–3.20 (m, 85H); 3.16–2.67 (m, 87H); 2.63–2.33 (m, 56H). ^13^C NMR (CDCl_3_, 100 MHz): δ 172.8, 172.6, 172.3, 169.1, 168.8, 160.8, 137.5, 135.2, 133.6, 129.1, 126.0, 125.0, 115.0, 52.1, 49.8, 47.7, 39.4, 38.9, 36.1, 31.5.

### 2.4. Synthesis of Boc-Lys(Boc) Dopamine (***7***) (Scheme 2)

A solution of Boc-Lys(Boc)-OH (0.20 g, 0.58 mmol) in CH_2_Cl_2_ (10.0 mL) was added dropwise to a solution of *N*-hydroxysuccinimide (79.7 mg, 0.69 mmol) and 1-ethyl-3-(3-dimethylaminopropyl)carbodiimide hydrochloride (EDC·HCl) (265.6 mg, 1.39 mmol) in CH_2_Cl_2_ (50.0 mL) in an ice bath. After stirring at 0 °C for 30 min, the ice bath was removed, and the reaction mixture was stirred at an ambient temperature for 6 h. A solution of dopamine hydrochloride (219.0 mg, 1.15 mmol) and Et_3_N (0.30 mL, 2.15 mmol) was added to the resulting mixture in a mixture of CH_2_Cl_2_ (10.0 mL) and MeOH (2.0 mL) at an ambient temperature and stirred for 1.5 d. After removing the solvent in vacuo, the residue was partitioned into CH_2_Cl_2_ (80.0 mL) and H_2_O (50.0 mL). The organic layer was washed with H_2_O (50.0 mL) twice, dried over MgSO_4_, and concentrated in vacuo to give a mixture. The mixture was purified by column chromatography (silica gel *ϕ* 2 cm × 12 cm, eluted by CH_2_Cl_2_:MeOH = 20:1 (200 mL), 15:1 (100 mL), 10:1 (200 mL)) to derive the desired compound as a white solid (187.0 mg, 67%, R_f_ = 0.25 (CH_2_Cl_2_:MeOH = 50:1)). ^1^H NMR (CDCl_3_, 400 MHz): δ 6.79 (d, 1H, *J* = 8.0 Hz); 6.66 (d, 1H, *J* = 2.0 Hz); 6.53 (d, 1H, *J* = 8.1 Hz); 6.33 (s, 1H); 5.33 (s, 1H); 4.80 (s, 1H); 3.97 (s, 1H); 3.59–3.47 (m, 1H); 3.40–3.28 (m, 1H); 3.00 (dd, 2H, *J*_1_ = 6.7, *J*_2_ = 13.3 Hz); 2.69–2.59 (m, 2H); 2.24–2.23 (m, 1H); 1.68–1.58 (m, 1H); 1.50–1.33 (m, 23H); 1.21–1.09 (m, 2H). ^13^C NMR (CDCl_3_, 100 MHz): δ 172.8, 156.8, 156.0, 144.44, 143.2, 130.8, 120.6, 116.0, 115.7, 80.3, 79.8, 54.6, 40.8, 40.1, 34.7, 32.3, 29.7, 28.5, 28.4, 22.5; MS (ESI) *m*/*z* calculated for C_24_H_39_N_3_NaO_7_ [M + Na]^+^: 504.2686; found: 504.2680.

**Scheme 2:**
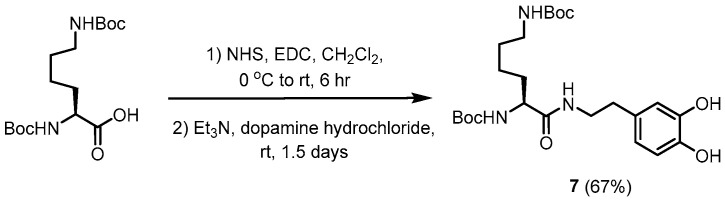
Synthetic scheme of Boc-Lys(Boc) dopamine (**7**).

### 2.5. Synthesis of NH_2_-βAla-OMe (***9***) (Scheme 3)

SOCl_2_ (2.67 g, 1.64 mL, 22.45 mmol) was added dropwise to a solution of β-alanine (2.00 g, 22.45 mmol) in CH_2_Cl_2_ (60.0 mL) by an addition funnel at 0 °C. The resulting mixture was stirred at 0 °C for 10 min. The ice bath was removed, and the resulting mixture was stirred at an ambient temperature overnight. The solvent was removed in vacuo. The resulting mixture was dissolved in a mixture of MeOH (10.0 mL) and CH_2_Cl_2_ (100.0 mL). Ethyl ether was added to the resulting solution until precipitation appeared. The resulting mixture was kept at 4 °C overnight. After filtration, the residue was washed with ether to derive the desired compound as a white solid (2.13 g, 92%). ^1^H NMR (CD_3_OD, 400 MHz): δ 3.73 (S, 3H); 3.23 (t, 2H, *J* = 6.64 Hz); 2.80 (t, 2H, *J* = 6.64 Hz). ^13^C NMR (CD_3_OD, 100 MHz): δ 171.4, 51.6, 35.4, 31.0. HRMS (ESI) *m*/*z* calculated for C_4_H_9_NO_2_ [M + H]^+^: 104.0708; found: 104.0708.

### 2.6. Synthesis of Boc-Phe-βAla-OMe (***11***) (Scheme 3)

Sodium hydroxide (970.0 mg, 24.22 mmol) was added to a solution of phenylalanine (2.00 g, 12.11 mmol) in a mixture of H_2_O and THF (1:1, 100.0 mL) The solution was stirred at rt for 20 min, and di-tert-butyl dicarbonate (5.28 g, 5.38 mL, 24.22 mmol) was slowly added to the mixture and stirred at an ambient temperature overnight. After removing the solvent in vacuo, the residues were dissolved in CH_2_Cl_2_ (80.0 mL) and extracted with H_2_O (20.0 mL × 3). The pH value of the aqueous layer was adjusted to 4–5 by 1*N* HCl_(aq)_. The mixture was stirred for 30 min and then extracted with CH_2_Cl_2_ (30.0 mL × 3). The organic layer was dried over MgSO_4_ and concentrated in vacuo to derive compound **10** as a colorless oil. EDC·HCl (2.22 g, 11.64 mmol) and HOBt (1.90 g, 11.64 mmol) were added to a solution of compound **10** (3.09 g, 11.64 mmol) in DMF (60.0 mL) at an ambient temperature and stirred for 20 min. Compound **9** (1.62 g, 11.64 mmol) and Et_3_N (1.18 g, 1.62 mL, 11.64 mmol) were added to the reaction mixture at an ambient temperature and stirred for 5 h. After removing the solvent in vacuo, the residue was partitioned into CH_2_Cl_2_ (100.0 mL) and H_2_O (20.0 mL). The organic layer was washed with H_2_O (20.0 mL) twice, dried over MgSO_4_, and concentrated in vacuo to give a mixture. The mixture was purified by column chromatography (silica gel, *ϕ* 2.5 cm × 14 cm, CH_2_Cl_2_:MeOH = 50:1 (500 mL), 30:1 (300 mL)) to derive the desired compound as a white solid (3.67 g, 90%, R_f_ = 0.25 (CH_2_Cl_2_:MeOH = 50:1)). ^1^H NMR (CDCl_3_, 400 MHz): δ 7.24–7.12 (m, 5H); 6.57 (t, 1H, *J* = 5.84 Hz); 5.30 (d,1H, *J* = 8.16 Hz); 4.28 (bs, 1H); 3.58 (s, 3H); 3.47–3.42 (m, 1H); 3.35–3.29 (m, 1H); 2.98 (d, 2H, *J* = 6.40 Hz); 2.42–2.32 (m, 2H); 1.35 (s, 9H). ^13^C NMR (CDCl_3_, 100 MHz): δ 172.7, 171.6, 155.5, 137.0, 129.5, 128.7, 127.0, 80.1, 56.1, 51.9, 39.1, 34.9, 33.8, 28.5. HRMS (ESI) *m*/*z* calculated for C_18_H_26_N_2_O_5_Na [M + Na]^+^: 373.1739; found: 373.1736.

### 2.7. Synthesis of Boc-Lys(Boc)-Phe-βAla-OMe (***12***) (Scheme 3)

Compound **11** (1.00 g, 2.85 mmol) was dissolved in 12*N* HCl and MeOH (1:2, 25.0 mL), and the reaction mixture was stirred for 1 h. The solvent was then removed in vacuo. The residue was dissolved in MeOH (25.0 mL) and neutralized by adding Et_3_N (381.4 mg, 0.4 mL, 3.77 mmol), and the solvent was subsequently evaporated to give deprotected **11**.

EDC·HCl (545.1 mg, 2.85 mmol) and HOBt (446.2 mg, 2.85 mmol) were added to a solution of Boc-Lys(Boc)-OH (989.0 mg, 2.85 mmol) in DMF (60.0 mL) at an ambient temperature and stirred for 20 min. Deprotected **11** and Et_3_N (865.5 mg, 1.20 mL, 8.55 mmol) were added in the reaction mixture at an ambient temperature and stirred overnight. After removing the solvent in vacuo, the residue was partitioned into CH_2_Cl_2_ (80.0 mL) and H_2_O (20.0 mL). The organic layer was washed with H_2_O (20.0 mL) twice, dried over MgSO_4_, and concentrated in vacuo to give a mixture. The mixture was purified by column chromatography (silica gel *ϕ* 2 cm × 12 cm, CH_2_Cl_2_:MeOH = 30:1 (400 mL), 20:1 (200 mL)) to derive the desired compound as a white solid (1.14 g, 69%, R_f_ = 0.30 (CH_2_Cl_2_:MeOH = 20:1)). ^1^H NMR (CDCl_3_, 400 MHz): δ 7.26–7.12 (m, 5H); 6.83 (s, 1H); 6.76 (s, 1H); 5.55 (s, 1H); 4.83 (s, 1H); 4.64 (q, 1H, *J*_1_ = 6.9, *J*_2_ = 10.8 Hz); 3.97 (s, 1H); 3.60 (s, 3H); 3.51–3.49 (m, 1H); 3.32–3.26 (m, 1H); 3.09–2.96 (m, 4H); 2.40–2.33 (m, 2H); 1.75–1.66 (m, 1H); 1.63–1.55 (m, 1H); 1.41 (s, 9H); 1.41–1.35 (m, 2H); 1.35 (s, 9H); 1.25–1.16 (m, 2H). ^13^C NMR (CDCl_3_, 100 MHz): δ 172.5, 172.2, 170.9, 156.7, 156.5, 136.7, 129.5, 128.8, 127.1, 80.4, 79.3, 55.3, 54.1, 51.9, 39.5, 38.3, 35.2, 33.8, 31.3, 29.9, 28.8, 28.5, 22.1. HRMS (ESI) *m*/*z* calculated for C_29_H_46_N_4_O_8_Na [M + Na]^+^: 601.3213; found: 601.3208.

### 2.8. Synthesis of Boc-Lys(Boc)-Phe-βAla Dopamine (***13***) (Scheme 3)

1*N* NaOH_(aq)_ (20.0 mL) was added to a solution of compound **12** (416.7 mg, 0.72 mmol) in EtOH (60.0 mL) and stirred at an ambient temperature for 25 min. Then, the pH value of the mixture was adjusted to 4–5 by 1*N* HCl_(aq)_. The mixture was stirred for 1 h, and the solvent was removed in vacuo. EDC·HCl (138.7 mg, 0.72 mmol) and HOBt (118.4 mg, 0.72 mmol) were added to a solution of the resulting residue in the DMF (60.0 mL) at an ambient temperature, and the mixture was stirred for 25 min. Dopamine hydrochloride (137.7 mg, 0.72 mmol) and Et_3_N (140.0 mg, 0.20 mL, 1.38 mmol) were added to the resulting solution and stirred for 2 d. After removing the solvent in vacuo, the residue was partitioned into CH_2_Cl_2_ (70.0 mL) and H_2_O (15.0 mL). The organic layer was washed with H_2_O (15.0 mL) twice, dried over MgSO_4_, and concentrated in vacuo to give a mixture which was purified by column chromatography (silica gel *ϕ* 2 cm × 14 cm, CH_2_Cl_2_:MeOH = 40:1 (300 mL), 30:1 (200 mL), 20:1 (100 mL), 10:1 (200 mL)) to derive the desired compound as a white solid (100.0 mg, yield: 60%, R_f_ = 0.20 (CH_2_Cl_2_:MeOH = 20:1)). ^1^H NMR (CDCl_3_, 400 MHz): δ 7.27–7.12 (m, 5H); 6.79 (d, 1H, *J* = 8.0 Hz); 6.72 (d, 1H, *J* = 2.0 Hz); 6.52 (q, 1H, *J*_1_ = 2.0, *J*_2_ = 8.0 Hz); 6.20 (s, 1H); 5.56 (s, 1H); 4.82 (s, 1H); 4.56–4.50 (m, 1H); 3.95 (s, 1H); 3.45–3.29 (m, 4H); 3.03 (d, 4H, *J* = 6.2 Hz); 2.66 (t, 2H, *J*_1_ = 6.5, *J*_2_ = 13 Hz); 2.24–2.23 (m, 1H); 2.18–2.17 (m, 1H); 2.05 (s, 1H); 1.72–1.65 (m, 1H); 1.62–1.53 (m, 1H); 1.45 (s, 9H); 1.38 (s, 9H); 1.24–1.17 (m, 2H). ^13^C NMR (CDCl_3_, 100 MHz): δ 173.0, 171.6, 171.2, 157.0, 156.6, 144.5, 143.4, 136.5, 131.4, 129.5, 128.9, 127.3, 120.7, 116.1, 115.7, 81.0, 79.7, 55.5, 54.6, 41.1, 39.4, 38.0, 36.5, 36.2, 34.7, 31.0, 30.0, 28.7, 28.5, 22.1. HRMS (ESI) *m*/*z* calculated for C_36_H_53_N_5_O_9_Na [M + Na]^+^: 722.3741; found: 722.3737.

**Scheme 3:**
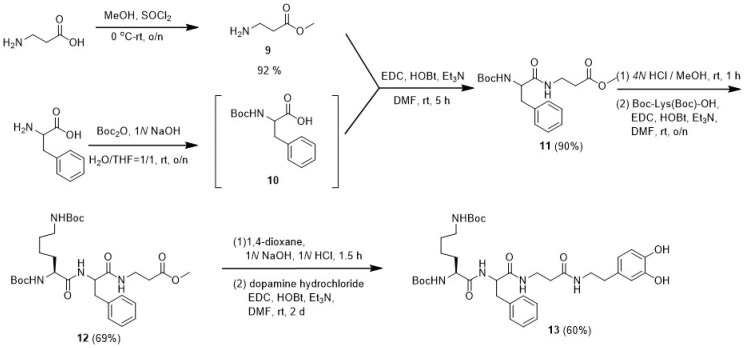
Synthetic scheme of Boc-Lys(Boc)-Phe-βAla dopamine (**13**).

### 2.9. Synthesis of (NH_2_-Lys((CPBA)_2_-Lys))_2_-Lys-CONH_2_ (***16***)

Rink amide resin (169 mg, loading ratio 0.3 mmol/g, 0.05 mmol) was subjected to swelling in DMF (1.5 mL) for 3 h. After the removal of DMF by filtration, the swelled resin was treated with 20% piperidine in DMF (1.5 mL) and agitated for 10 min twice to remove the Fmoc group. After filtration, the activated resin was washed with DMF (3.0 mL) and CH_2_Cl_2_ (3.0 mL) three times each. The solution of Fmoc-Lys(Fmoc)-OH (105 mg, 0.175 mmol) and HBTU (68 mg, 0.175 mmol) was dissolved in 5% NMM in DMF (1.5 mL) and pre-mixed for 15 min. The mixture was then added to the activated resin and agitated for 1 h for the coupling of the first residue. After the removal of the solvent by filtration, the loaded resin was washed with DMF (3.0 mL) and CH_2_Cl_2_ (3.0 mL) three times each. Piperidine (20%) was added to the loaded resin in DMF (1.5 mL) and agitated for 10 min twice to remove the Fmoc group. The resin was washed with DMF (3.0 mL) and CH_2_Cl_2_ (3.0 mL) three times each for the following synthesis.

For the incorporation of the second residue, a solution of Boc-Lys(Fmoc)-OH (165 mg, 0.35 mmol) and HBTU (135 mg, 0.35 mmol) was dissolved in 5% NMM in DMF (1.5 mL) and was subjected to the same procedure as the coupling of the first residue, with a 1.5 h coupling time. For the incorporation of the third residue, Fmoc-Lys(Fmoc)-OH (208 mg, 0.35 mmol) and HBTU (132 mg, 0.35 mmol) in 5% NMM in DMF (1.5 mL) was subjected to the same procedure of the coupling of the first residue, with a 2 h coupling time. A solution of 4-carboxyphenylboronic acid (117 mg, 0.7 mmol) and PyBOP (365 mg, 0.7 mmol) in 5% NMM in DMF (1.5 mL) was added to the resulting resin, and the resulting mixture was shaken for an additional 14 h. After removing the solution by filtration, the resulting mixture was washed with DMF (3.0 mL) and CH_2_Cl_2_ (3.0 mL) three times each. The crude product was cleaved from the resin with 1 mL of TFA/H_2_O (95:5) for 2 h.

After filtration, the resulting filtrate was precipitated with diethyl ether and kept at 0 °C for 30 min. After centrifugation twice (6000 rpm, 15 min), the supernatant was decanted to collect the product. This procedure was repeated to collect the final product (64.3 mg, 87%).

^1^H NMR (D_2_O, 400 MHz): δ 7.59 (d, *J* = 8.0 Hz, 8H); 7.54 (d, *J* = 8.0 Hz, 4H); 7.47 (d, *J* = 8.0 Hz, 4H); 4.37 (t, *J* = 8.0 Hz, 2H); 4.18 (t, *J* = 8.0 Hz, 1H); 3.95 (t, *J* = 8.0 Hz, 1H); 3.82 (t, *J* = 8.0 Hz, 1H); 3.36–3.26 (m, 4H); 3.21–3.05 (m, 6H); 1.89–1.21 (m, 30H). ^13^C NMR (CD_3_OD, 100 MHz): δ 176.18, 174.80, 170.49, 170.18, 163.17, 162.88, 138.43, 136.94, 136.26, 134.93, 129.97, 127.51, 127.18, 55.94, 54.50, 54.23, 40.42, 39.62, 32.77, 32.52, 32.10, 30.14, 29.81, 29.54, 24.47, 24.14, 23.03, 22.64. Mass (MALDI–TOF, DHB/**16**/NaI = 10/1/1) *m*/*z* calculated for C_86_H_91_B_4_N_11_O_25_Na_1_ [M+Na]+: 1744.6; found: 1744.7.

### 2.10. General Procedure of Boronate Ester Formation Assay [30]

A solution of boronic acid derivatives (50.0 mg) in toluene (2.0 mL) and EtOH (1.0 mL) was added dropwise to a solution of catechol derivatives in toluene (3.0 mL) at an ambient temperature. The reaction mixture was then heated to reflux for 5 d with a Dean–Stark apparatus. The mixture was filtered and washed by toluene (1.0 mL), and the solid part was collected for further analysis of TBAF coordination.

### 2.11. General Procedure of N→B Coordination Boronate Ester Assay

Compound **7** (66.0 mg, 0.060 mmol) was dissolved in toluene (3.0 mL) at an ambient temperature. Compound **6** (50.0 mg, 0.004 mmol) was dissolved in toluene (2.0 mL) and EtOH (1.0 mL). The solution of compound **6** was added dropwise to the solution of compound **7** at an ambient temperature. Piperidine (2 equivalents) was added to the reaction mixture and refluxed for 5 d with a Dean–Stark apparatus. The solvent was removed in vacuo, and the product was collected for TBAF coordination analysis.

Dopamine hydrochloride (18.2 mg, 0.096 mmol) was dissolved in toluene (3.0 mL) at an ambient temperature. Compound **16** (30.0 mg, 0.024 mmol) was dissolved in toluene (2.0 mL) and EtOH (1.0 mL). The solution of compound **16** was added dropwise to the solution of dopamine hydrochloride at an ambient temperature and refluxed for 5 d with a Dean–Stark apparatus. The solvent was removed in vacuo, and the product was collected for TBAF coordination analysis.

### 2.12. TBAF Coordination Experiment

The samples were dissolved in DMSO-*d*_6_. TBAF was added, and immediately, NMR spectra were acquired. All the experiments were performed in quartz NMR tubes.

## 3. Results and Discussion

**Figure 2:**
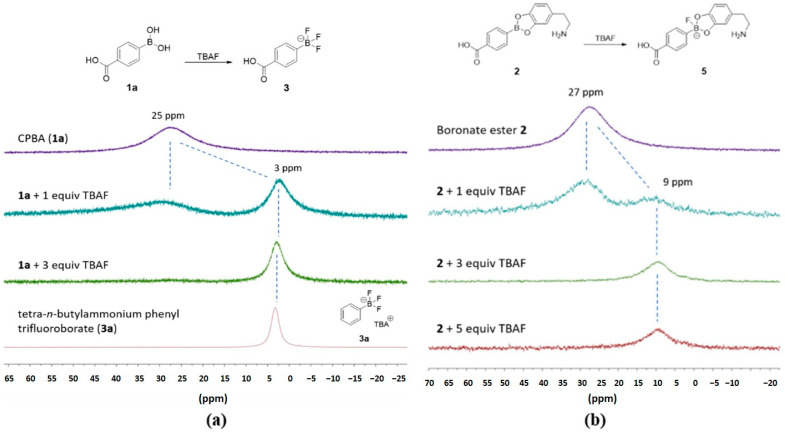
^11^B NMR spectra of (**a**) boronic acid **1a**, with TBAF, and tetra-*n*-butylammonium phenyl trifluoroborate (**3a**); (**b**) boronate ester **2** with TBAF. All spectra were acquired in DMSO-*d*_6_.

This study was the first to attempt to collect the ^11^B NMR spectra of both 4-carboxyphenyl boronic acid (CPBA, **1a**) and corresponding boronate esters (**2**), which was synthesized and characterized according to the literature [30]. The ^11^B NMR spectra of **1a** and **2** generated similar signals between 25 and 30 ppm. To enhance the difference in electron densities of the boron centers, tetrabutylammonium fluoride (TBAF) was gradually added to a solution of **1a**, and their ^11^B NMR spectra were collected. The results clearly show that the amount of TBAF has a positive correlation with the intensity of the signal at 3 ppm and a negative correlation with the intensity of the signal at 25 ppm (Figure 2a). Fluoride interacts with boron, resulting in a splitting signal in ^11^B NMR spectra. However, no splitting signal was observed, possibly due to the broad signals of **1a** and **2** in the ^11^B NMR spectra. Two trifluoroborates were referenced: tetra-n-butylammonium phenyl trifluoroborate (**3a**) (Figure 2a), which showed a signal at 3.3 ppm, and potassium (4-carboxyphenyl)trifluoroborate (**3b**) (Figure S9e), which presented a signal at 2.2 ppm. Both signals were observed without splitting. Therefore, we assumed the newly formed signal at 3.5 ppm represented (4-carboxyphenyl)trifluoroborate (**3**). On the contrary, while boronate ester **2** was treated with TBAF, the resulting fluorinated derivatives afforded an ^11^B NMR signal at 9.4 ppm (Figure 2b). We believe that monofluorinated boronate was generated. This observation aligns with that of literature reports [26,31]. These results suggest that adding fluoride (3 equivalents) to boronic acids and boronate esters gave the corresponding trifluoroborates and fluoroboronate esters, which generated two signals around 3 ppm and 9 ppm, respectively. These two signals represented boronic acids and boronate esters.

**Figure 3:**
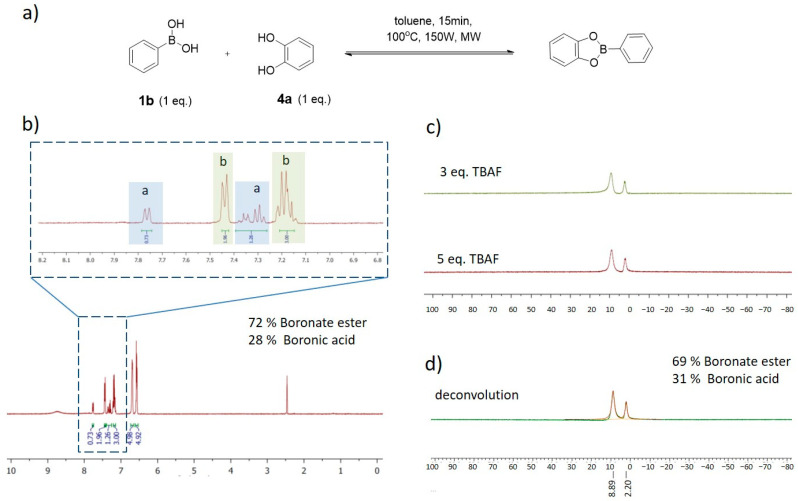
(**a**) Reaction scheme of boronate ester bond formation between catechol and PBA. (**b**) ^1^H NMR spectra of catechol (1 equivalent) reacted with PBA (1 equivalent ) with the irradiation of a microwave (150 W) for 30 min; the inserted figure is the region from 6.8 to 8.2 ppm, wherein signal **a** represents the proton on the aromatic ring of **1b**, and signal **b** represents the proton on the PBA aromatic ring of boronate ester. (**c**) ^11^B NMR spectra of catechol (1 equivalent) reacted with PBA (1 equivalent) for 30 min, with the irradiation of a microwave (150 W) with 3 and 5 equivalents of TBAF. (**d**) Deconvolution spectra of the spectrum in (**c**). All spectra were acquired in DMSO-*d*_6_.

The ratio of boronic acids to boronate esters was determined from the integral of the ^1^H NMR spectrum before the addition of fluoride ions. The equilibrium in the presence of fluoride ions was assessed using the ^11^B NMR spectrum. Two sets of results were compared to evaluate the influence of fluoride ions on the equilibrium. The synthesized boronate ester was created by mixing equal amounts of phenylboronic acid (PBA, **1b**) and catechol (**4**) (Figure 3a). In the absence of TBAF in the analyte, a signal between 7 and 8 ppm, which corresponds to the aromatic signal of PBA in the ^1^H NMR spectrum, indicated that the ratio of boronic acid to boronate ester is 28% to 72% (Figure 3b). This result was calculated by the integral of the aromatic ring on PBA, shifting before and after boronate ester formation [32]. In Figure 3b, signal **a** (Figure 3b, in blue region) gives the integral of **1b**, which is in the boronic acid form of the analyte; signal **b** (Figure 3b, in green region) gives the integral of PBA, which is in the boronate ester form of the analyte. The amount of boronate ester formation was calculated by the two ratios of the blue and green region (Figure 3b). After adding TBAF, the resulting ^11^B NMR spectra indicated that the ratio of boronic acid and boronate ester was 31% to 69%. (Figure 3d) This observation suggests that the TBAF titration did not significantly alter the ratio of boronic acid and boronate ester. Therefore, the ^11^B NMR spectra of the TBAF titration could determine the ratio of boronic acid and boronate ester.

**Figure 4:**
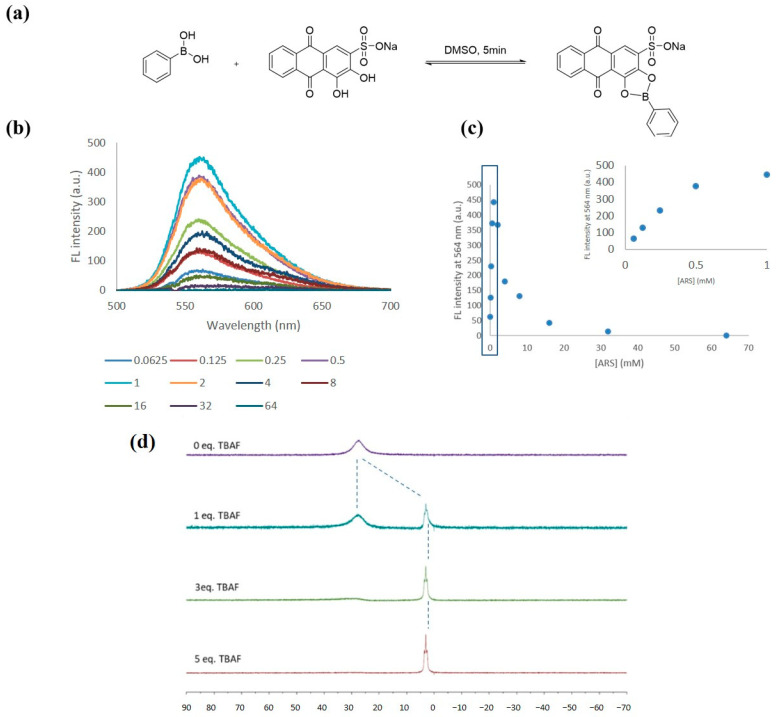
(**a**) Reaction scheme of boronate ester bond formation between ARS and PBA. (**b**) Fluorescent spectra. (**c**) Fluorescent intensity at 564 nm of 0.0625–64 mM of ARS mixed with 64 mM of PBA for a 5 min incubation time; the inserted figure shows the range from 0.0625 to 1 mM of ARS mixed with 64 mM of PBA. (**d**) ^11^B NMR spectra of 1 mM of ARS mixed with 64 mM of PBA for a 5 min incubation time with 1, 3, 5 equivalents of TBAF. All spectra were acquired in DMSO-*d*_6_.

Alternatively, titrating with alizarin red S (ARS) is a common method to assess the binding affinity between boronic acids and diols [33]. This method is based on the formation of an ARS–boronate ester, which leads to fluorescence emission. Compared with the ^11^B NMR method, a high concentration of boronic acid is required due to the low sensitivity of ^11^B NMR. In the TBAF titration experiment, 64 mM of phenylboronic acid (PBA) was mixed with 64 mM of acid red dye (ARS) to produce a signal at 10.0 ppm, indicating the presence of a boronate ester. However, the linear range of ARS concentration extends up to 0.6 mM for fluorescence emission at 564 nm with 64 mM of PBA. This observation suggests a possible self-quench for the solution of 64 mM of PBA, while the concentration of ARS is more than 1 mM (Figure 4b,c). For this experiment, 1 mM of PBA was used instead. Unfortunately, in the presence of ARS (64 mM), no clear boronate ester was observed in the ^11^B NMR spectrum after the TBAF titration experiments. Therefore, the ARS binding experiment could not provide the amount of boronate ester at a concentration that would yield a significant ^11^B NMR signal.

**Figure 5:**
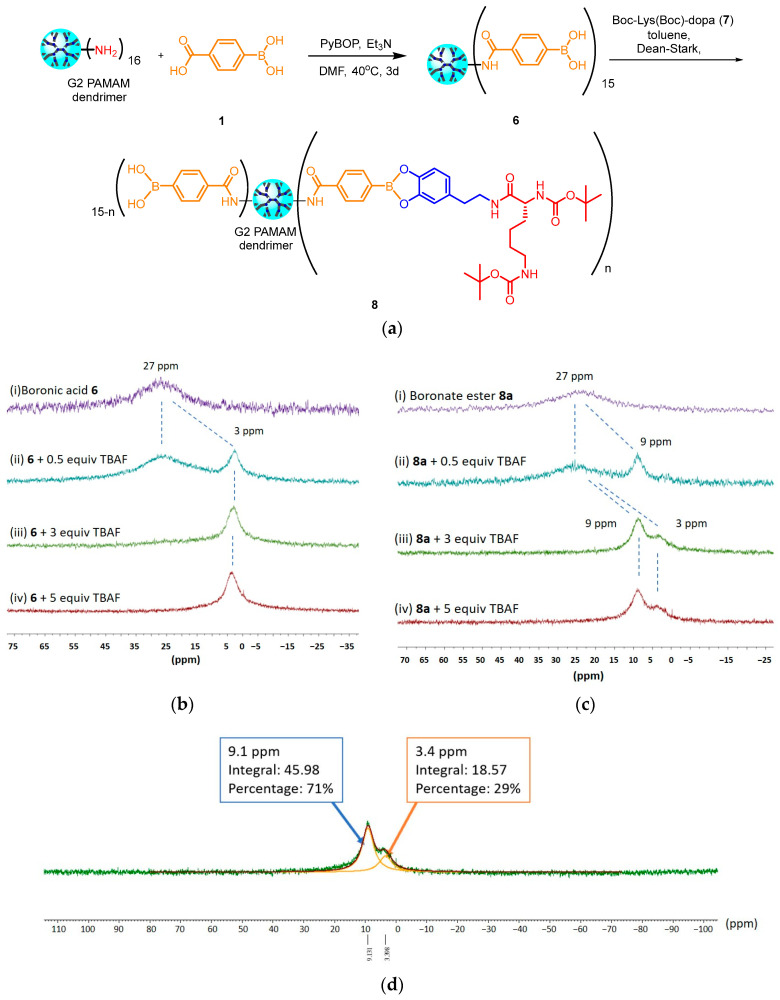
(**a**) Synthetic scheme of boronic acid **6** and boronate ester **8**. ^11^B NMR spectra of (**b**) boronic acid **6** and (**c**) boronate ester **8a** (*n* = 11) with different equivalents of TBAF. (**d**) Deconvolution spectra of the spectrum in (**c-iv**). All spectra were acquired in DMSO-*d*_6_.

Since the addition of fluoride ions did not alter the equilibrium between the boronic acid and boronate ester, this study shifted its focus to the identification of macromolecular boronate esters, whereby (G:2)-*dendri*-PAMAM-(CPBA)_15_ (**6**) was prepared, characterized [34], and subjected to a TBAF–^11^B NMR experiment (Figure 3a). With the presence of TBAF (3 equivalents), a resonance at 3.8 ppm was recorded, and the signal at 25.6 ppm was diminished (Figure 5b). Thereafter, the boronate ester was prepared between **6** and dopamine-Lys(Boc)-Boc (**7**). A mixture of **7** and **6** (3:1) was added to TBAF (1 equivalent), and two resonances at 9.1 and 25.6 ppm were recorded in a ^11^B NMR spectrum. With two more equivalents of TBAF, the resonance at 25.6 ppm was completely diminished, and a signal at 9.1 ppm remained alongside a new signal at 3.4 ppm (Figure 5c). Accordingly, these two resonances represented boronic acid and boronate ester. Because of the overlapping of the two signals at 3.4 and 9.1 ppm, it was difficult to clearly distinguish the integrals and give the ratio of the two boron derivatives. The deconvolution method was employed to isolate the signals of the two molecules and obtain their respective integrals for the ratio of boronic acids and boronate esters. The deconvolution results indicate that the ratio of the integrals of the resonances at 3.4 and 9.1 is 29% to 71% (Figure 5d). The number of boronate esters on **8a** was 11. Therefore, **8a** was (G:2)-*dendri*-PAMAM-[(NH_2_)(CPBA)_4_(Boc-Lys(Boc)-dopa boronate ester)_11_]. This result clearly shows that this method can distinguish boronic acids and boronate esters and determine their ratios in macromolecules.

After successfully monitoring in situ, the investigation shifted to exploring the impact of separation, which is a potential method for collecting pure boronate ester. However, separating boronate esters from the product mixture can lead to a shift in equilibrium and a loss of boronate esters, potentially damaging them [35]. Because of the complicated equilibrium between boronic acids and boronate esters, it is difficult to evaluate the damage caused by separation procedures. To study this issue, boronic acid-decorated dendrimers with multiple peripheral boronic acids are a potential tool. In this research, we investigated the influence of precipitation and size-exclusion chromatography (SEC) on boronate esters and evaluated their impairment of boronates using a fluorinated method. We mixed dendrimer **6** with peptide **7** and heated it with a Dean–Stark apparatus to obtain the crude boronate-modified dendrimer **8**. Upon adding 3 equivalents of TBAF, the resulting dendrimer showed a sole signal of around 9 ppm, indicating no free boronic acid on the dendrimers (entry 1, Table 1). We then subjected dendrimer **8** to either Sephadex^®^ LH-20 SEC or precipitation in EtOH/ether. The dendrimers were collected and treated with TBAF. The resulting mixture was monitored using ^11^B NMR spectra, and 59% and 72% of boronate ester in the dendrimers remained after SEC chromatography and precipitation, respectively (entries 2 and 3, Table 1, Figures S12 and S13). It is worth mentioning that the chromatography took only 2 h, while precipitation required 1 day, twice. Despite the less time needed for chromatography, more boronate esters were lost than during precipitation. Presumably, the ratio of the two components kept changing during the column chromatography, and the Sephadex^®^ LH-20 shifted the equilibrium to **6**. Based on this result, precipitation damages fewer boronate esters. The TBAF coordination method identified the stability of boronate esters in macromolecules during separation.

Instead of using a Dean–Stark apparatus within a week of the reaction time, various amounts of **7** was added for the formation of boronate esters in hours. To monitor the progression of the preparation, this TBAF–^11^B NMR method was applied to in situ track the formation of boronate esters. When 1 equivalent of **7** was used, 57% of the boronate was obtained in 5 days (Table 2, entry 1). However, when 2 equivalents of **7** were used, over 99% of the boronate was obtained (Table 2, entries 1–2). Remarkably, the reaction time was reduced to one day, and only 45% of the boronate ester was observed, with 2 equivalents of **7** (Table 2, entry 3).

**Figure 6:**
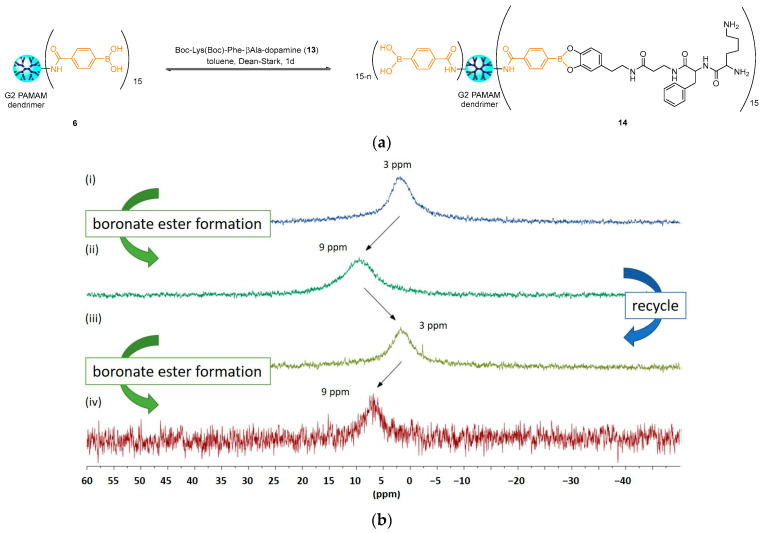
(**a**) Interconversion between boronic acid **6** and corresponding boronate ester **14**. (**b**) ^11^B NMR spectra of recycle cycles for boronic acid containing dendrimer with TBAF (3 equivalents), (**i**) boronic acid containing dendrimer **6**, (**ii**,**iv**) boronate ester formation of **14**, and (**iii**) recycling of boronic acid containing dendrimer **6**. Conditions of boronate ester formation: 1:5 equivalents of **6** to **13**; dialysis in pH = 6. All spectra were acquired in DMSO-*d*_6_.

This TBAF–^11^B NMR method was applied to monitor the reversible formation of boronate esters. Boronate esters were known to be sensitive to pH environments, and low pH values lead to the dissociation of boronate esters. This pH value-dependent boronate formation has been widely applied to pH-responsive materials [36]. However, tracking their formation and degradation is rarely reported. To monitor the recovery of boronic acid by the TBAF–^11^B NMR method, **6** was mixed with Boc-Lys(Boc)-Phe-βAla dopamine (**13**) (5 equivalents) in one day to convert all the boronic acids to corresponding (G:2)-*dendri*-PAMAM-[(NH_2_) (Boc-Lys(Boc)-Phe-βAla-dopa boronate ester)_15_] (**14**). The boronate **14** was added to TBAF, and its ^11^B NMR spectrum was acquired (Figure 6(bi)). Thereafter, the mixture was dialyzed under pH = 6 for 1 day, twice, to hydrolyze the boronate ester to the boronic acid as the solo boron analog (Figure 6(bii)). The recovering **6** was subjected to the same reaction condition as the first boronate ester formation, which gave the same fully functionalized boronate ester **14** (Figure 6(biii)). The ^11^B NMR spectrum of the mixture at each stage showed a clear signal shift between the boronic acids and boronate esters. This result demonstrates that this method is useful for following the equilibrium between boronic acids and corresponding boronate esters.

**Figure 7:**
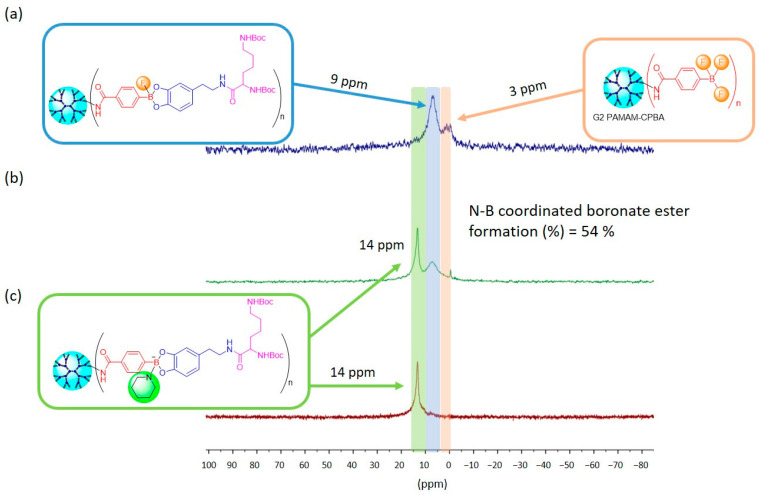
^11^B NMR spectra of N–B-coordinated boronate ester **15** with TBAF (3 equivalents), with different equivalents of piperidine added; (**a**) without piperidine, (**b**) piperidine (1 equivalent), (**c**) piperidine (2 equiv); percentage of N–B-coordinated boronate ester **15** formation was calculated by deconvolution. All spectra were acquired in DMSO-*d*_6_.

With similar encouraging results, this method has been applied to detect N–B-coordinated boronate esters, which was reported as stable boronate ester derivatives. The presence of amine can increase the ratio of boronate esters [37]. Among various Lewis bases, amines are general and efficient chemicals for generating a dative nitrogen–boron coordination to stabilize boronate esters [38,39,40]. However, the in situ monitoring of nitrogen–boron coordinations remains a challenge for monitoring the formation of N–B-coordinated boronate esters. Piperidine (1 equivalent) was added to the mixture of **7** and **6** (1:1), and the ^11^B–NMR spectrum of the resulting mixture with TBAF (3 equivalents) gave two signals at 13.8 ppm and 7.6 ppm (Figure 7). The signal at 13.8 ppm represented the amine-coordinated boronate ester, which matches the reported literature [40]. Remarkably, the signal at 13.8 ppm remains with an intake of up to three equivalents of TBAF (Figure 7c). Therefore, ^11^B NMR offers clear evidence for the presence of N–B-coordinated boronate esters from boronic acids and boronate esters when adding TBAF. When the piperidine was increased to two equivalents, no signal representing boronic acid was observed (Figure 7c). With more piperidine (2 equivalents), one equivalent of catechol derivatives was enough for the completed formation of boronate esters. On the contrary, 3 equivalents of catechol (**7**) are necessary for the formation of boronate esters at 71%. This result proves the critical role of N–B coordination for the formation of boronate esters. Alongside our aforementioned results, the ^11^B NMR spectra provided clear evidence for distinguishing boronic acids, boronate esters, and amine-coordinated boronate esters by the TBAF–^11^B NMR method (Figure 7).

**Figure 8:**
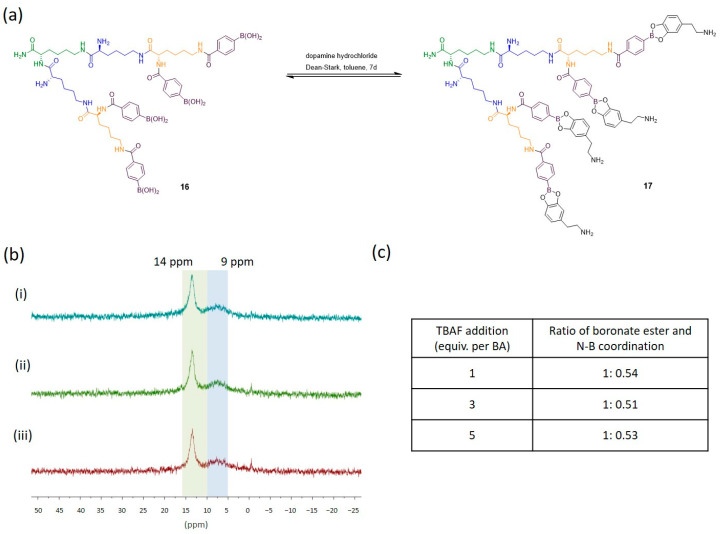
(**a**) Structure of N–B-coordinated boronate ester **17**. (**b**) ^11^B NMR spectra of N–B-coordinated boronate ester **17** with (**i**) 1, (**ii**) 3, and (**iii**) 5 equivalents of TBAF and the deconvolution spectra. (**c**) Table of the results of the deconvolution.

This TBAF–^11^B NMR method was used to identify the boronate ester formation of boronic acid-decorated lysine dendrimer (**16**) with dopamine. Dendrimer **16** was prepared and identified based on the literature [41]. After treating with dopamine (1 equivalent), the resulting complex **17** was added to the TBAF (1 equivalent), and its ^11^B NMR spectrum only showed two signals at 7.4 and 13.4 ppm, but no signal was identified at around 3 ppm. This observation suggests no boronic acids remained when adding 1 equivalent of dopamine (Figure 8(bi)). On the contrary, 2 equivalents of catechols were necessary for the complete formation of boronates in the previous example (Table 2). For this observation, the intramolecular N–B coordination was believed to stabilize the boronate esters from boronic acids. For calculating the ratio of amine-coordinated boronate esters, **17** was treated with 1, 3, or 5 equivalents of TBAF, and their resulting complexes showed similar spectra (Figure 8b). The ratio of integrals indicated that the ratio of boronate esters and amine-coordinated derivatives is 1: 0.5, which suggests that 35% of N–B coordination was present in this product’s mixture. This example clearly demonstrates the potential of this TBAF–^11^B NMR method to identify and to calculate the ratio of boronic acids, boronate esters, and amine-coordinated boronate esters.

## 4. Conclusions

In summary, the ^11^B NMR spectra with the fluoride-coordinated method were developed to in situ monitor the formation of boronate esters in macromolecules. Boronic acids and boronate esters react with TBAF to yield trifluoroborates and fluoroboronate esters. Remarkably, adding fluoride ions did not alter the equilibrium between the boronic acid and boronate ester. Amine-coordinated boronate esters remain unaffected under similar conditions. As a result, three different boronic acid derivatives exhibit distinct signals at 3, 9, and 14 ppm in the ^11^B NMR spectra after being treated with TBAF. For the first time, the equilibrium among boronic acids, boronate esters, and amine-coordinated boronate esters was measured in situ. Despite the additional TBAF required for measurements, this method could monitor the progression of the formation of boronate esters and amine-coordinated derivatives in a mixture of boronic acids and the compound with *cis*-diols.

The results of this investigation suggest that precipitation is a better purification method than chromatography. Although both methods hydrolyzed the boronate esters, 72% of the boronate esters remained after precipitation. This method was also used to identify the ratio of intermolecular amine-coordinated boronate esters. Based on the result of the TBAF–^11^B NMR analysis, 1 equivalent of dopamine was enough to convert all boronic acids to boronate esters in dendrimer **16**. A further spectrum analysis suggested that 35% of the N–B coordination was found in the products. This study sheds light on the mechanistic investigation and application of dynamic covalent boronate esters in macromolecules.

## Figures and Tables

**Table 1:** Ratio of boronate ester **8a** after purification ^a^.

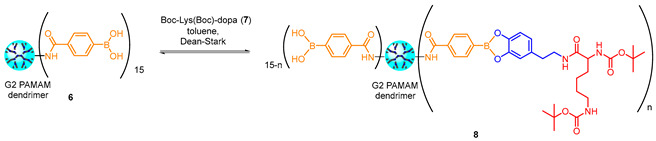				
Entry	Status	Solvent	Manipulation Time	Normalized Boronate Ester (%) ^b^
1	Before purification	-	-	100
2	After SEC purification	MeOH	2 h	59
3	After precipitation	EtOH/ether	1 day (twice)	72

^a^: the mixture was treated with TBAF (3 equivalents), and the spectra were recorded in a solution of DMSO-*d*_6_. ^b^. The ratio was calculated by each integral, which was estimated by a deconvolution of the ^11^B NMR spectra of the mixtures. The ^11^B NMR spectra of the original and the results afwter deconvolution are shown in Figures S12 and S13.

**Table 2:** The ratio of boronate ester **8** with various amounts of **7** ^a^.

Entry	7 (Equivalents)	Reaction Time (d)	Integral Ratio at 3 ppm:9 ppm	Boronate Ester (%) ^b^	n
1	1	5	0.74:1	57	9
2	2	5	1	>99	15
3	2	1	1.2:1	45	7

^a^: the mixture was treated with TBAF (3 equivalents), and the spectra were recorded in a solution of DMSO-*d*_6_. ^b^. The ratio was calculated by each integral, which was estimated by a deconvolution of the ^11^B NMR spectra of the mixtures.

## Data Availability

All data reported in this paper are contained within the manuscript and Supplementary Materials.

## Supplementary Materials

The following supporting information can be downloaded at: https://www.mdpi.com/article/10.3390/polym16233258/s1, Scheme S1: Synthetic scheme of boronate ester conjugated G2 PAMAM dendrimer. Figure S1: ^1^H NMR (A), ^13^C NMR (B) and ^11^B NMR (C) spectra of compound **2** recorded in DMSO-*d*_6_ at 300 K. Figure S2: ^1^H NMR (A) and ^13^C NMR (B) spectra of compound **6** recorded in CD_3_OD at 300 K. Figure S3: ^1^H NMR (A) and ^13^C NMR (B) spectra of compound **7** recorded in CDCl_3_ at 300 K. Figure S4: ^1^H NMR (A) and ^13^C NMR (B) spectra of compound **9** recorded in CD_3_OD at 300 K. Figure S5: ^1^H NMR (A) and ^13^C NMR (B) spectra of compound **11** recorded in CDCl_3_ at 300 K. Figure S6: ^1^H NMR (A) and ^13^C NMR (B) spectra of compound **12** recorded in CDCl_3_ at 300 K. Figure S7: ^1^H NMR (A) and ^13^C NMR (B) spectra of compound **13** recorded in CDCl_3_ at 300 K. Figure S8: ^1^H NMR (A) and ^13^C NMR (B) spectra of compound **16** recorded in D_2_O at 300 K. Figure S9: ^11^B NMR spectra of (a) boronic acid **1a**, (b) **1a** titrated with TBAF (1 equiv), (c) **1a** with TBAF (3 equiv), (d) tetra-*n*-butylammonium phenyl trifluoroborate (**3a**), (e) potassium (4-carboxyphenyl)trifluoroborate (**3b**). All spectra were acquired in DMSO-*d*_6_. Figure S10. ^11^B NMR spectra of 1 mM of ARS mixed with (a) 16 mM (b) 4 mM of PBA in 5 min incubation time with 3, 5, 10 equiv of TBAF. All spectra were acquired in DMSO-*d*_6_. Figure S11. ^11^B NMR spectra of 1 mM of ARS reacted with 4 mM of PBA in 30 min, 150 W by microwave with 3, 5, 10 equiv of TBAF. All spectra were acquired in DMSO-*d*_6_. Figure S12: ^11^B-NMR spectra of boronate ester **8a** with TBAF (3 equiv), (a) before puri-fication, (b) after Sephadex^®^ LH-20 column purification, (c) after precipitation by EtOH/ether. All spectra were acquired in DMSO-*d*_6_. Figure S13: Deconvolution result for Figure S12; ^11^B-NMR spectra of boronate ester **8a** with TBAF (3 equiv), (a) before purification, (b) after Sephadex^®^ LH-20 column purification, (c) after precipitation by EtOH/ether. All spectra were acquired in DMSO-*d*_6_. Figure S14: ^11^B-NMR spectra of boronate ester **8** at various conditions of equiv of catechol **7** and reaction time. All the sample were added TBAF (3 equiv), (a) **7** to **6** (2:1), reaction time: 1 d (Table 2, entry 1), (b) equiv of **7** to **6** (1:1) (Table 2, entry 2), reaction time: 5 d, (c) equiv of **7** to **6** (2:1) (Table 2, entry 3), reaction time: 5 d. All spectra were acquired in DMSO-*d*_6_. Figure S15: Deconvolution result for Figure S14; ^11^B-NMR spectra of boronate ester **8** with TBAF (3 equiv), (a) **7** to **6** (2:1), reaction time: 1 day (Table 2, entry 1), (b) equiv of **7** to **6** (1:1) (Table 2, entry 2), reaction time: 5 d, (c) equiv of **7** to **6** (2:1) (Table 2, entry 3), reaction time: 5 d. All spectra were acquired in DMSO-*d*_6_.

## References

[1] Bull S.D., Davidson M.G., van den Elsen J.M.H., Fossey J.S., Jenkins A.T.A., Jiang Y.-B., Kubo Y., Marken F., Sakurai K., Zhao J. (2013). Exploiting the Reversible Covalent Bonding of Boronic Acids: Recognition, Sensing, and Assembly. Acc. Chem. Res..

[2] Suzuki Y., Kusuyama D., Sugaya T., Iwatsuki S., Inamo M., Takagi H.D., Ishihara K. (2020). Reactivity of boronic acids toward catechols in aqueous solution. J. Org. Chem..

[3] Heleg-Shabtai V., Aizen R., Orbach R., Aleman-Garcia M.A., Willner I. (2015). Gossypol-cross-linked boronic acid-modified hydrogels: A functional matrix for the controlled release of an anticancer drug. Langmuir.

[4] Cai B., Luo Y., Guo Q., Zhang X., Wu Z. (2017). A glucose-sensitive block glycopolymer hydrogel based on dynamic boronic ester bonds for insulin delivery. Carbohydr. Res..

[5] Tarus D., Hachet E., Messager L., Catargi B., Ravaine V., Auzély-Velty R. (2014). Readily prepared dynamic hydrogels by combining phenyl boronic acid-and maltose-modified anionic polysaccharides at neutral pH. Macromol. Rapid Commun..

[6] Chen Y., Tang Z., Zhang X., Liu Y., Wu S., Guo B. (2018). Covalently cross-linked elastomers with self-healing and malleable abilities enabled by boronic ester bonds. ACS Appl. Mater. Interfaces.

[7] Deng C.C., Brooks W.L., Abboud K.A., Sumerlin B.S. (2015). Boronic acid-based hydrogels undergo self-healing at neutral and acidic pH. ACS Macro Lett..

[8] Li Z., Yu R., Guo B. (2021). Shape-Memory and Self-Healing Polymers Based on Dynamic Covalent Bonds and Dynamic Noncovalent Interactions: Synthesis, Mechanism, and Application. ACS Appl. Bio Mater..

[9] Cho S., Hwang S.Y., Oh D.X., Park J. (2021). Recent progress in self-healing polymers and hydrogels based on reversible dynamic B–O bonds: Boronic/boronate esters, borax, and benzoxaborole. J. Mater. Chem. A.

[10] Zheng J., Oh X.Y., Ye E., Chooi W.H., Zhu Q., Loh X.J., Li Z. (2023). Self-healing polymer design from dynamic B–O bonds to their emerging applications. Mater. Chem. Front..

[11] Liu B., Li J., Zhang Z., Roland J.D., Lee B.P. (2022). pH responsive antibacterial hydrogel utilizing catechol–boronate complexation chemistry. Chem. Eng. J..

[12] Zhao N., Yuan W. (2023). Antibacterial, conductive nanocomposite hydrogel based on dextran, carboxymethyl chitosan and chitosan oligosaccharide for diabetic wound therapy and health monitoring. Int. J. Biol. Macromol..

[13] Marco-Dufort B., Tibbitt M.W. (2019). Design of moldable hydrogels for biomedical applications using dynamic covalent boronic esters. Mater. Today Chem..

[14] Cai Y., Fu X., Zhou Y., Lei L., Wang J., Zeng W., Yang Z. (2023). A hydrogel system for drug loading toward the synergistic application of reductive/heat-sensitive drugs. J. Control. Release.

[15] Ren Q., Cheng Y., Lv J. (2023). Boronate building blocks for intracellular protein delivery. Adv. Healthc. Mater..

[16] Theodosis-Nobelos P., Charalambous D., Triantis C., Rikkou-Kalourkoti M. (2020). Drug conjugates using different dynamic covalent bonds and their application in cancer therapy. Curr. Drug Deliv..

[17] Seidi F., Jenjob R., Crespy D. (2018). Designing smart polymer conjugates for controlled release of payloads. Chem. Rev..

[18] Sun X., Chapin B.M., Metola P., Collins B., Wang B., James T.D., Anslyn E.V. (2019). The mechanisms of boronate ester formation and fluorescent turn-on in ortho-aminomethylphenylboronic acids. Nat. Chem..

[19] Smith M.K., Northrop B.H. (2014). Vibrational properties of boroxine anhydride and boronate ester materials: Model systems for the diagnostic characterization of covalent organic frameworks. Chem. Mater..

[20] Sharma B., Bugga P., Madison L.R., Henry A.-I., Blaber M.G., Greeneltch N.G., Chiang N., Mrksich M., Schatz G.C., Van Duyne R.P. (2016). Bisboronic acids for selective, physiologically relevant direct glucose sensing with surface-enhanced Raman spectroscopy. J. Am. Chem. Soc..

[21] Vancoillie G., Hoogenboom R. (2016). Synthesis and polymerization of boronic acid containing monomers. Polym. Chem..

[22] Cambre J.N., Sumerlin B.S. (2011). Biomedical applications of boronic acid polymers. Polymer.

[23] Babcock L., Pizer R. (1980). Dynamics of boron acid complexation reactions. Formation of 1:1 boron acid-ligand complexes. Inorg. Chem..

[24] Pizer R., Tihal C. (1992). Equilibria and reaction mechanism of the complexation of methylboronic acid with polyols. Inorg. Chem..

[25] Wan W.M., Cheng F., Jäkle F. (2014). A Borinic Acid Polymer with Fluoride Ion-and Thermo-responsive Properties that are Tunable over a Wide Temperature Range. Angew. Chem. Int. Ed..

[26] Bentley J.N., Caputo C.B. (2019). Substituent effects on the Lewis acidity of 4, 6-di-tert-butylchatechol boronate esters. Tetrahedron.

[27] Oehlke A., Auer A.A., Jahre I., Walfort B., Rüffer T., Zoufalá P., Lang H., Spange S. (2007). Nitro-substituted stilbeneboronate pinacol esters and their fluoro-adducts. Fluoride ion induced polarity enhancement of arylboronate esters. J. Org. Chem..

[28] Yuan M.S., Du X., Liu Z., Li T., Wang W., Anslyn E.V., Wang J. (2018). Di-(2-picolyl)-N-(2-quinolinylmethyl) amine-Functionalized Triarylboron: Lewis Acidity Enhancement and Fluorogenic Discrimination Between Fluoride and Cyanide in Aqueous Solution. Chem. Eur. J..

[29] DiCesare N., Lakowicz J.R. (2002). New sensitive and selective fluorescent probes for fluoride using boronic acids. Anal. Biochem..

[30] Christinat N., Croisier E., Scopelliti R., Cascella M., Röthlisberger U., Severin K. (2007). Formation of Boronate Ester Polymers with Efficient Intrastrand Charge-Transfer Transitions by Three-Component Reactions. Eur. J. Inorg. Chem..

[31] Reetz M.T., Niemeyer C.M., Harms K. (1991). Crown Ethers with a Lewis Acidic Center: A New Class of Heterotopic Host Molecules. Angew. Chem. Int. Ed. Engl..

[32] Marciasini L.D., Richard J., Cacciuttolo B., Sartori G., Birepinte M., Chabaud L., Pinet S., Pucheault M. (2019). Magnesium promoted autocatalytic dehydrogenation of amine borane complexes: A reliable, non-cryogenic, scalable access to boronic acids. Tetrahedron.

[33] Springsteen G., Wang B. (2002). A detailed examination of boronic acid–diol complexation. Tetrahedron.

[34] Tsai C.-H., Tang Y.-H., Chen H.-T., Yao Y.-W., Chien T.-C., Kao C.-L. (2018). A selective glucose sensor: The cooperative effect of monoboronic acid-modified poly (amidoamine) dendrimers. Chem. Commun..

[35] Asokan K., Shaikh K.M., Tele S.S., Chauthe S.K., Ansar S., Vetrichelvan M., Nimje R., Gupta A., Gupta A.K., Sarabu R. (2018). Applications of 2, 2, 2 trifluoroethanol as a versatile co-solvent in supercritical fluid chromatography for purification of unstable boronate esters, enhancing throughput, reducing epimerization, and for additive free purifications. J. Chromatogr. A.

[36] He L., Fullenkamp D.E., Rivera J.G., Messersmith P.B. (2011). pH responsive self-healing hydrogels formed by boronate–catechol complexation. Chem. Commun..

[37] He C., Dong J., Xu C., Pan X. (2023). N-Coordinated Organoboron in Polymer Synthesis and Material Science. ACS Polym. Au.

[38] Li H., Liu Y., Liu J., Liu Z. (2011). A Wulff-type boronate for boronate affinity capture of cis-diol compounds at medium acidic pH condition. Chem. Commun..

[39] Wang H., Grohmann C., Nimphius C., Glorius F. (2012). Mild Rh (III)-catalyzed C–H activation and annulation with alkyne MIDA boronates: Short, efficient synthesis of heterocyclic boronic acid derivatives. J. Am. Chem. Soc..

[40] Christinat N., Scopelliti R., Severin K. (2004). A new method for the synthesis of boronate macrocycles. Chem. Commun..

[41] Liao Y., Chan Y.-T., Molakaseema V., Selvaraj A., Chen H.-T., Wang Y.-M., Choo Y.-M., Kao C.-L. (2022). Facile Solid-Phase Synthesis of Well-Defined Defect Lysine Dendrimers. ACS Omega.

