# Supplementary material for: A 11B-NMR Method for the In Situ Monitoring of the Formation of Dynamic Covalent Boronate Esters in Dendrimers
Source: Polymers (Basel). 2024 Nov 23;16(23):3258. doi: 10.3390/polym16233258 (PMC11644288; doi:10.3390/polym16233258)
Supplement: Supplementary file 1 [file polymers-16-03258-s001.zip › polymers-3304590-supplementary.pdf]

Supplementary Materials

A ^{11}B-NMR method for the in situ monitoring of the formation of dynamic covalent boronate esters in dendrimers

Yi-Wen Yao [1], Ching-Hua Tsai [1], Chih-Yi Liu [1], Fang-Yu Wang [1], Sodio C. N. Hsu [1], Chun-Cheng Lin [1,2] , Hui-Ting Chen [3] and Chai-Lin Kao [1,4,5,6,7,8,*] [1] Department of Medicinal and Applied Chemistry, Kaohsiung Medical University, Kaohsiung 807, Taiwan; wendyyao.chem@gmail.com (Y.-W.Y.); crime93@yahoo.com.tw (C.-H.T.);
u109021119@gap.kmu.edu.tw (C.-Y.L.); u109021115@gap.kmu.edu.tw (F.-Y.W.);
sodiohsu@kmu.edu.tw (S.C.N.H.); cclin66@mx.nthu.edu.tw (C.-C.L.)

[2] Department of Chemistry, National Tsing Hua University, Hsinchu 300, Taiwan

[3] Department of Pharmacy, National Yang Ming Chiao Tung University, Taipei 112, Taiwan; htchen1969@nycu.edu.tw

[4] Department of Medical Research, Kaohsiung Medical University Hospital, Kaohsiung 807, Taiwan

[5] Drug Development and Value Creation Research Center, Kaohsiung Medical University, Kaohsiung 807, Taiwan.

[6] Center for Tropical Medicine and Infectious Disease Research, Kaohsiung Medical University, Kaohsiung 807, Taiwan.

[7] Department of Chemistry, National Sun Yat-sen University, Kaohsiung 804, Taiwan.

[8] College of Professional Studies, National Pingtung University of Science and Technology, Pingtung 912, Taiwan

[*] Correspondence: clkao@kmu.edu.tw

Content

I. Preparation of chemicals in this investigation 2

II. Analytical data of chemicals 3

III. ^{11}B NMR spectra and deconvolution results of dendrimers with TBAF 12

I. Preparation of chemicals in this investigation

Scheme S1. Synthetic scheme of boronate ester conjugated G2 PAMAM dendrimer

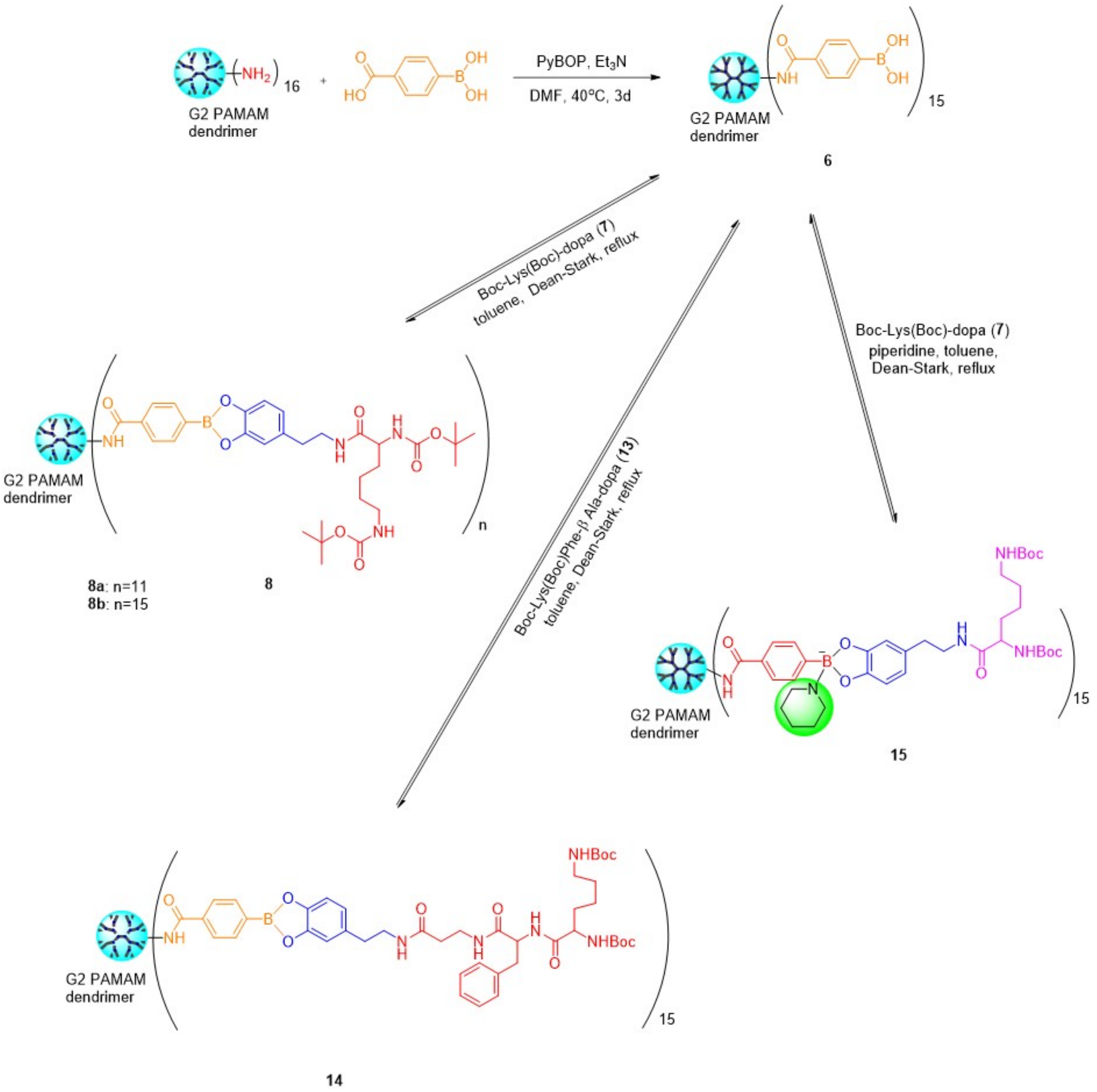

II. Analytical data of chemicals

(A)

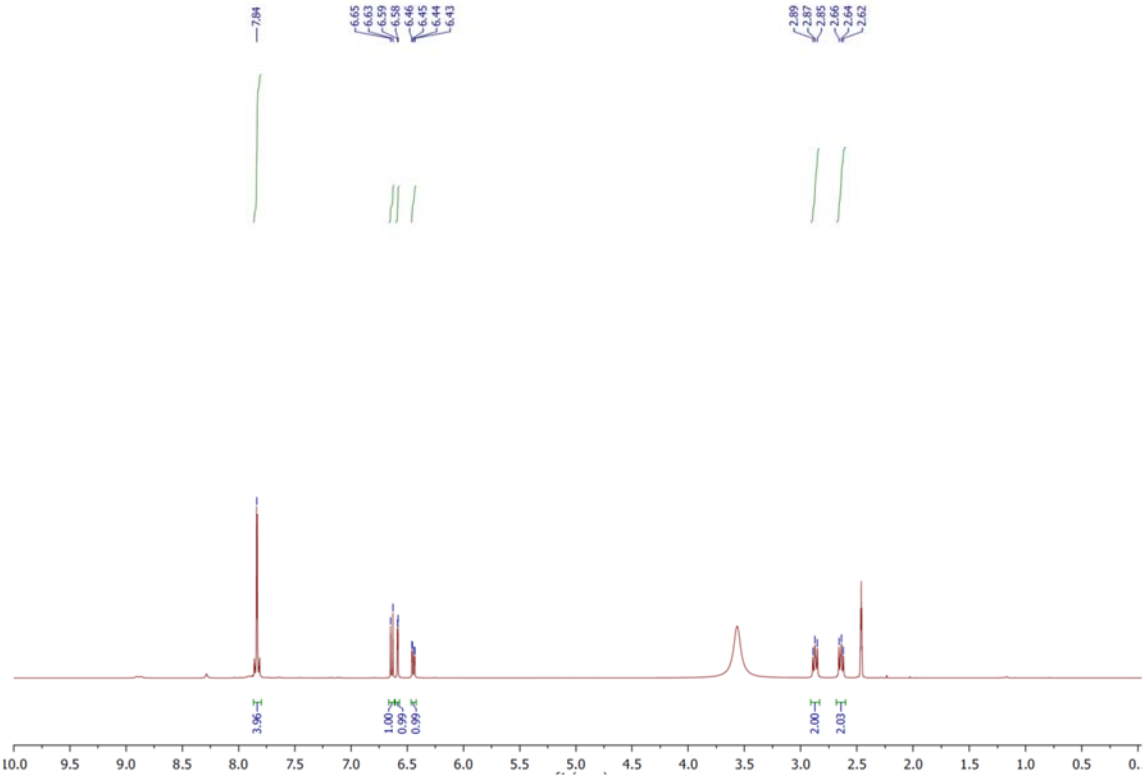

(B)

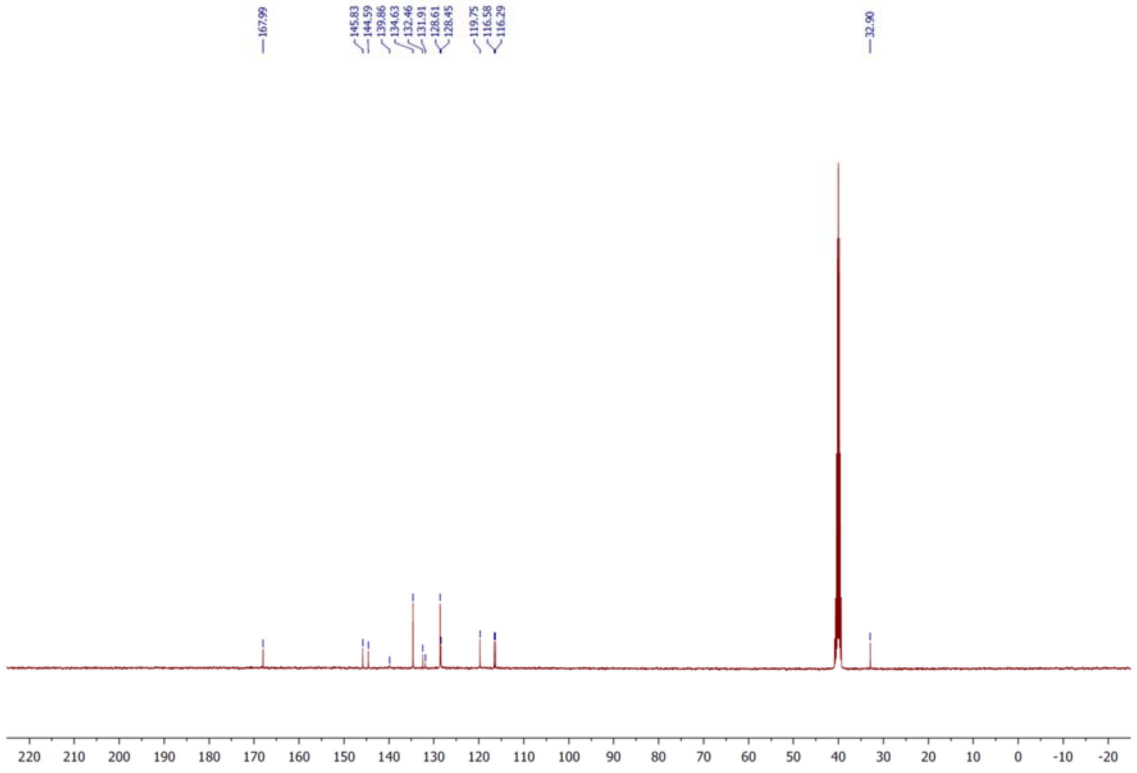

(C)

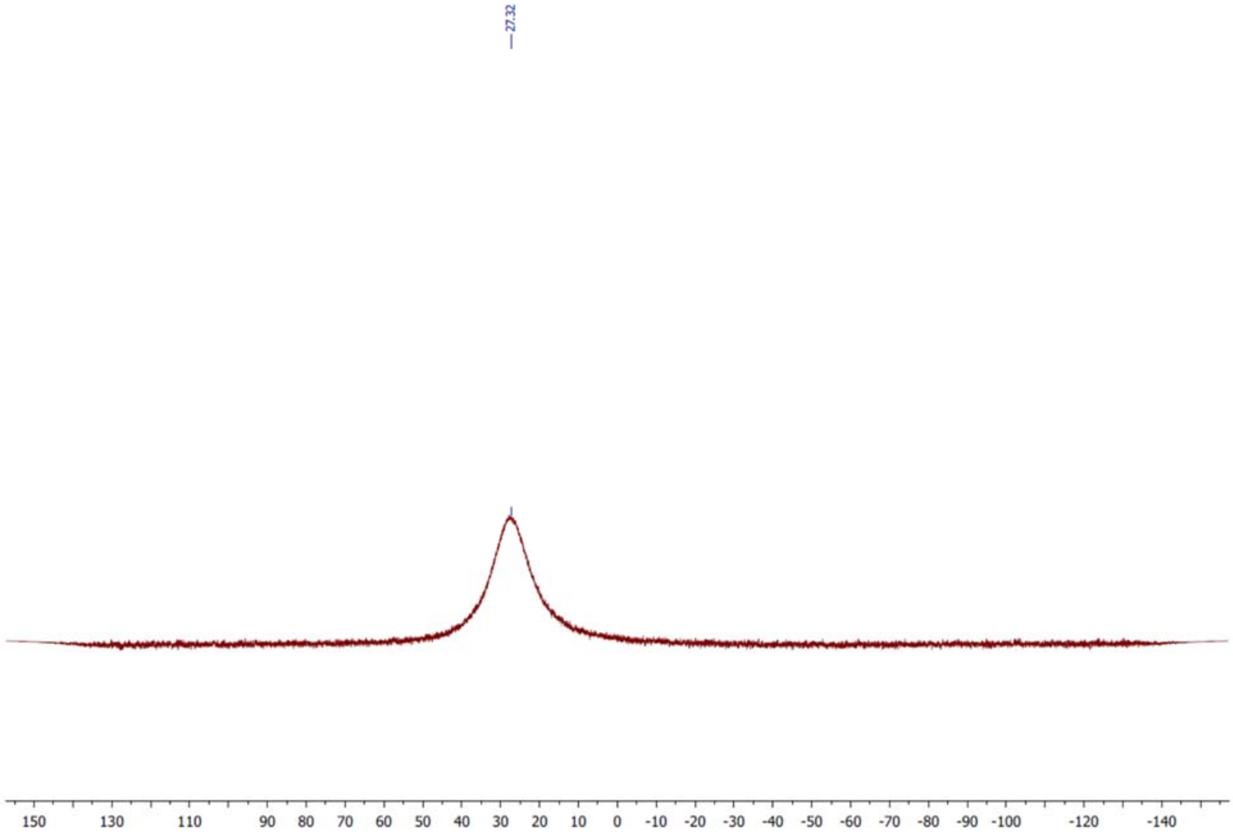

Figure S1: ^{1}H NMR (A), ^{13}C NMR (B) and ^{11}B NMR (C) spectra of compound **2** recorded in DMSO-d_6 at 300K

(A)

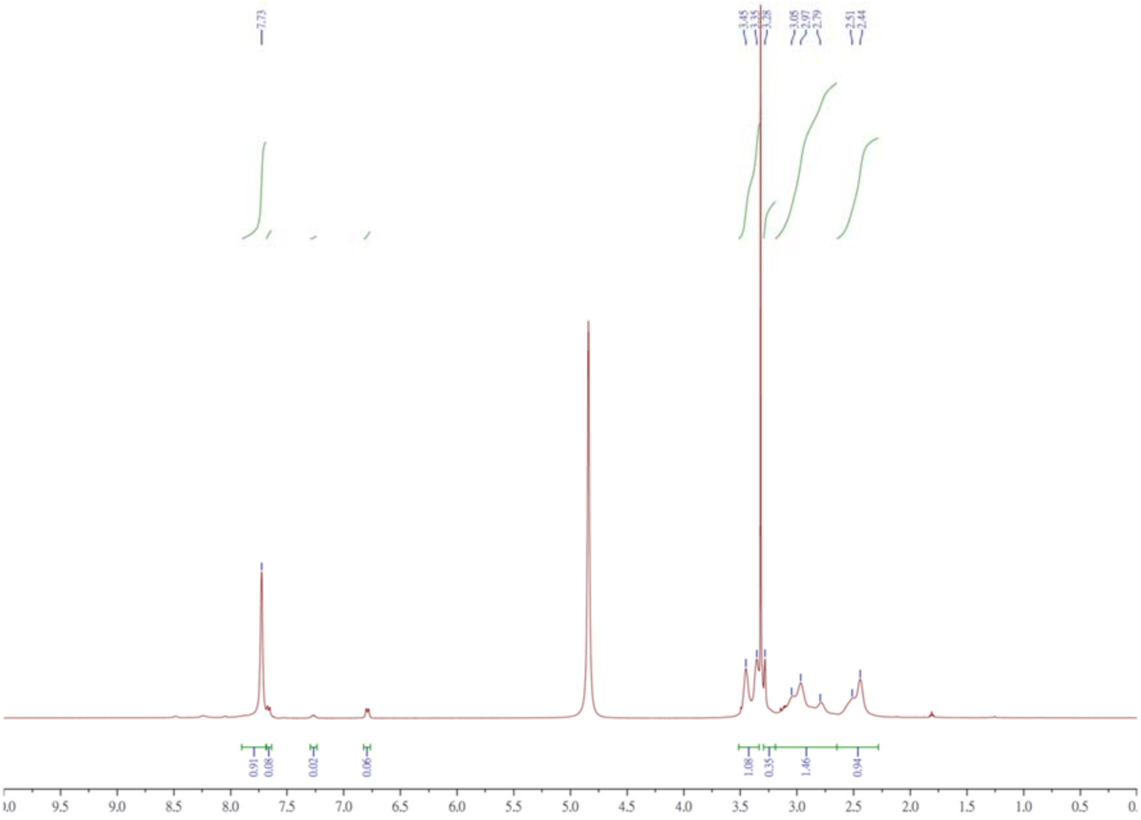

(B)

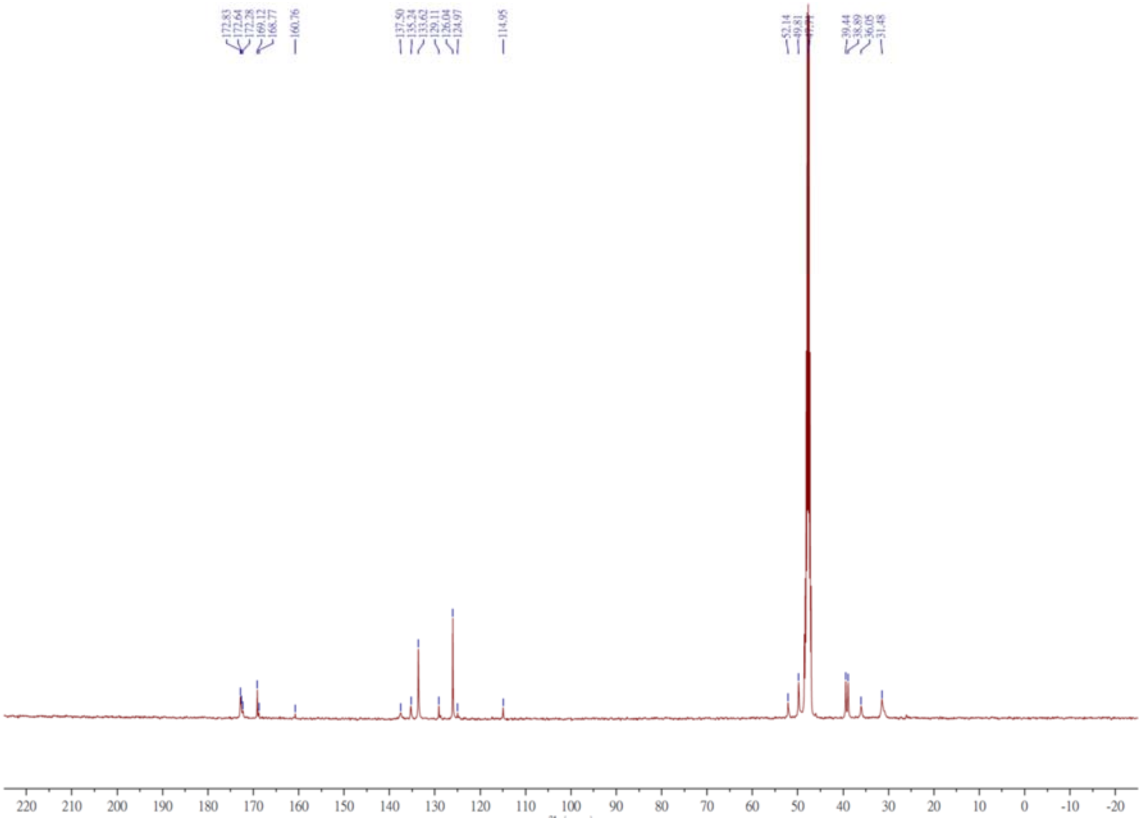

Figure S2: ^{1}H NMR (A) and ^{13}C NMR (B) spectra of compound **6** recorded in CD_3OD at 300K

(A)

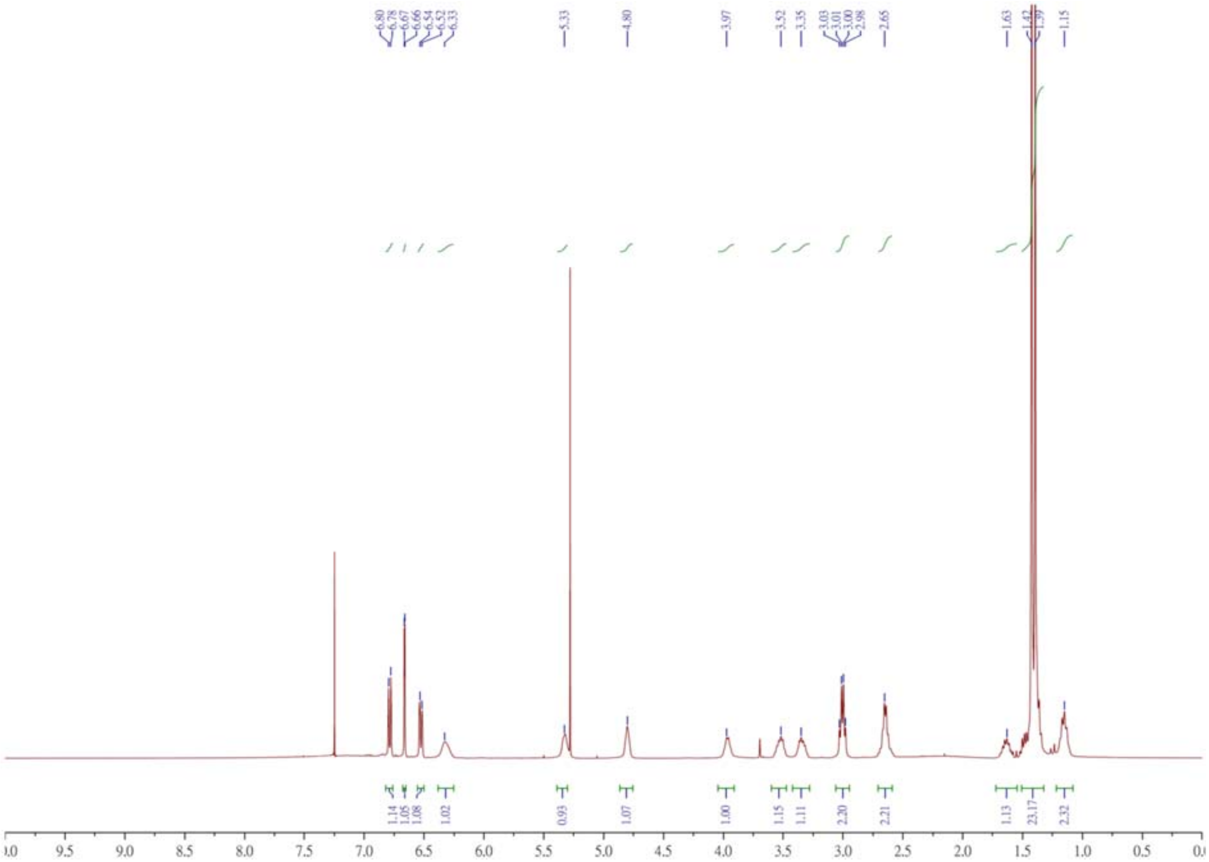

(B)

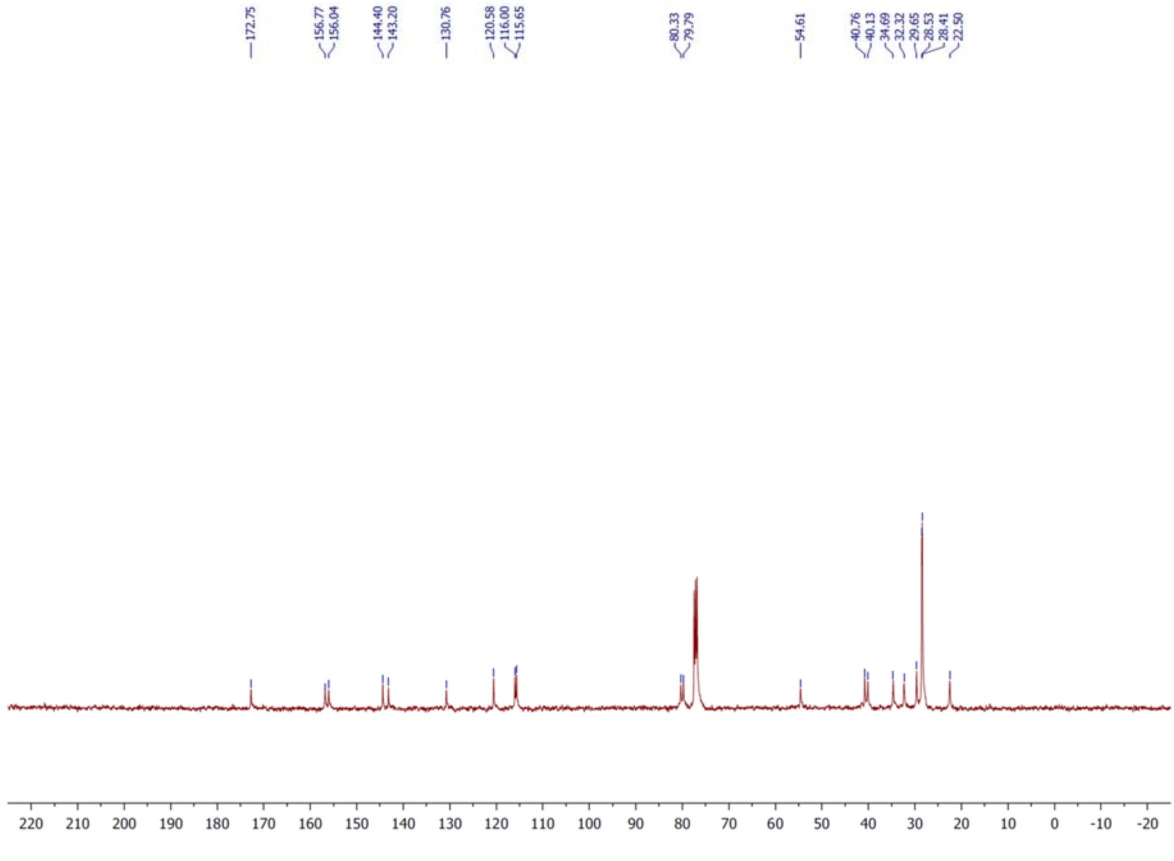

Figure S3: ^{1}H NMR (A) and ^{13}C NMR (B) spectra of compound **7** recorded in $CDCl_3$ at 300K

(A)

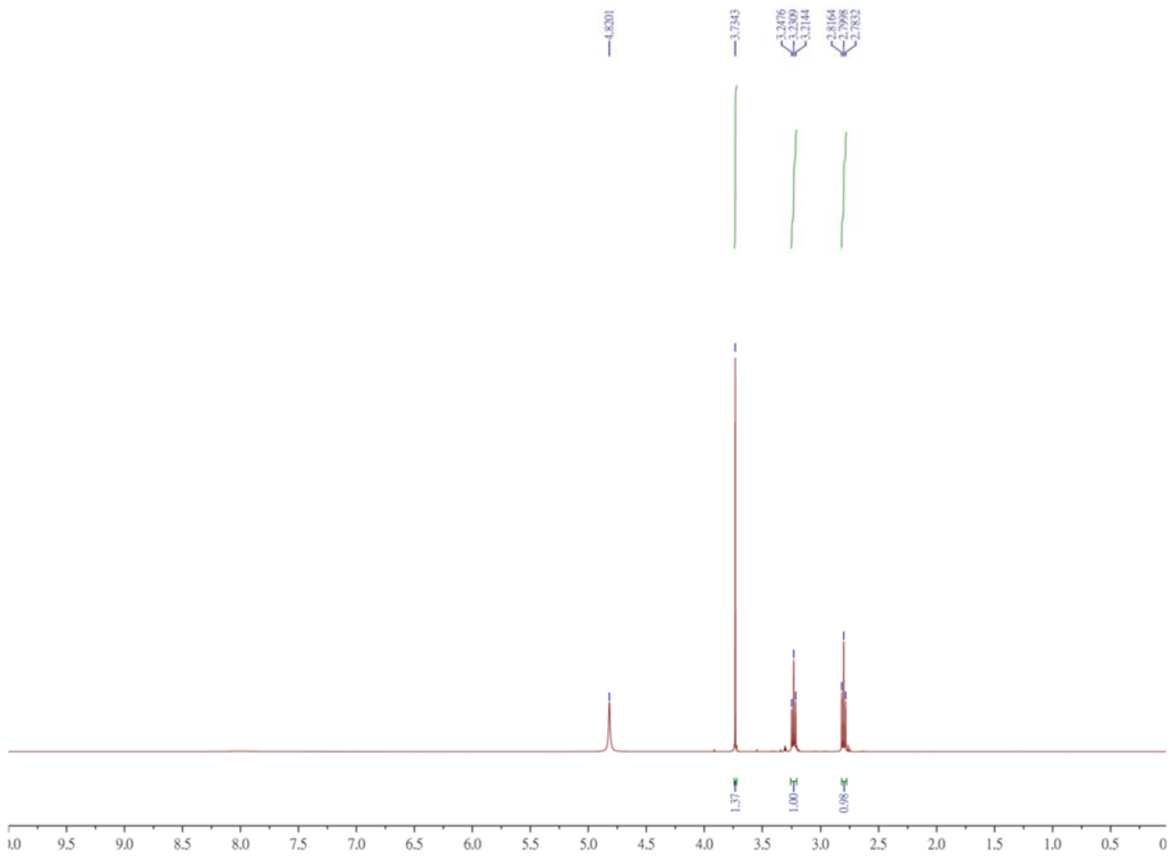

(B)

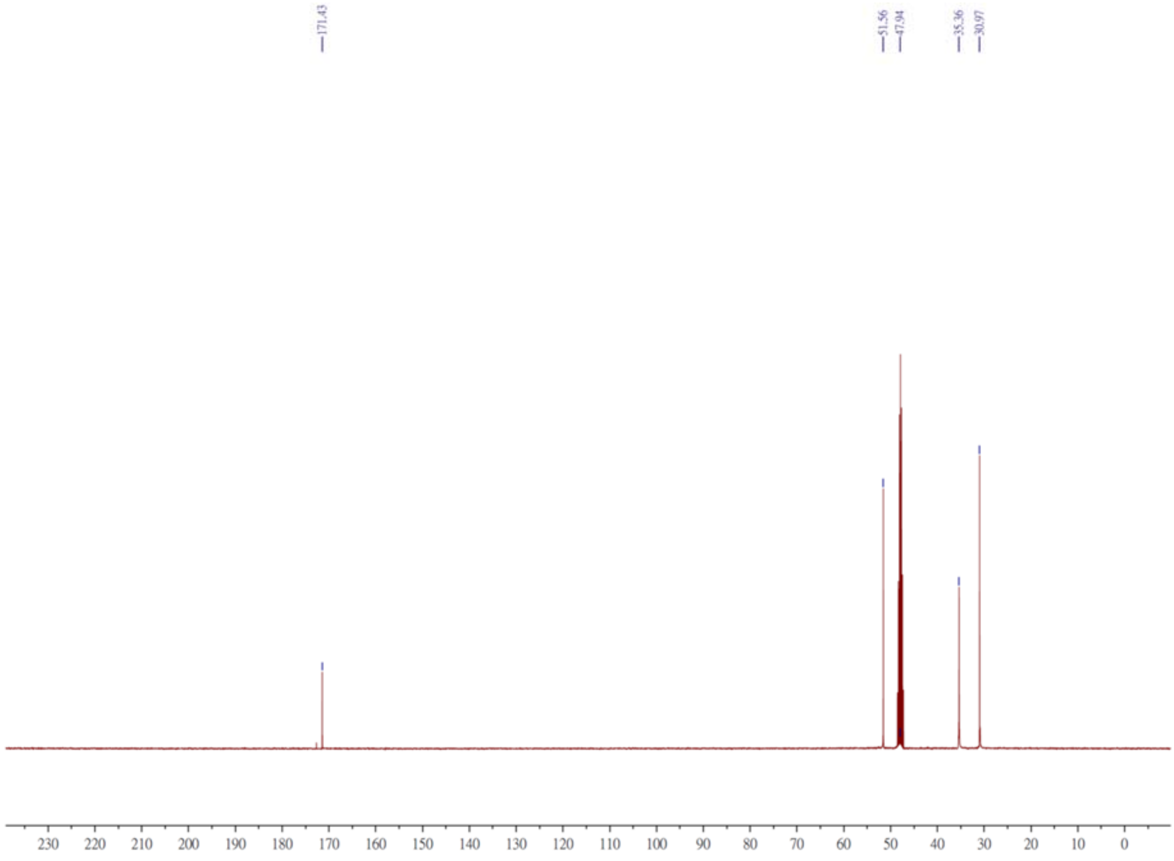

Figure S4: ^{1}H NMR (A) and ^{13}C NMR (B) spectra of compound **9** recorded in CD_3OD at 300K

(A)

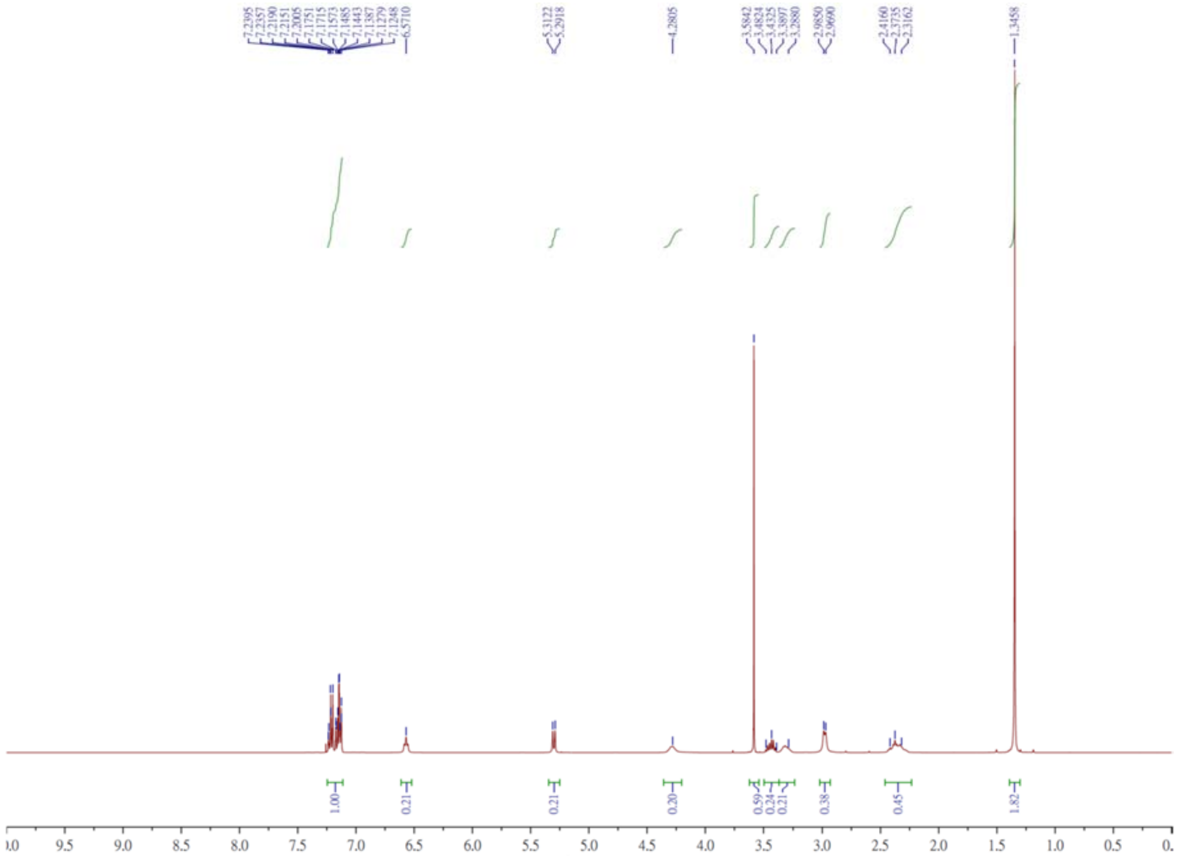

(B)

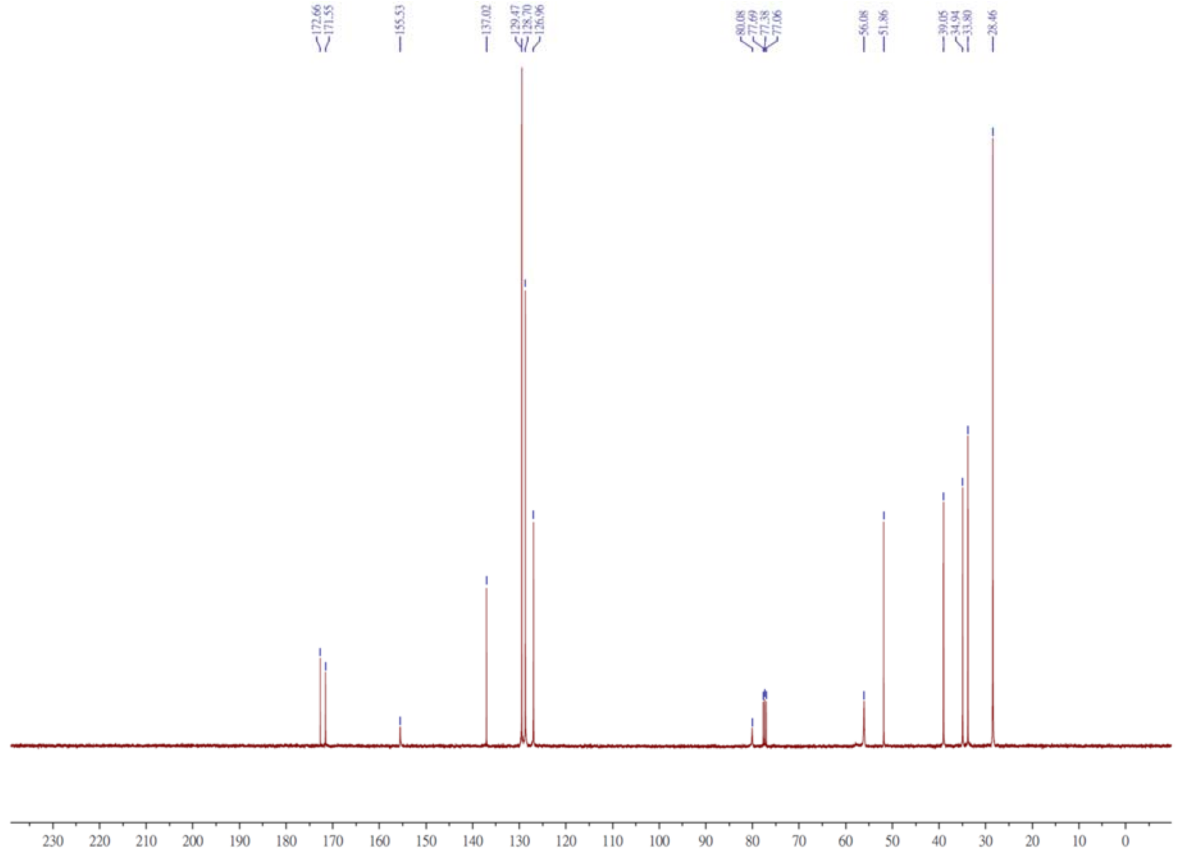

Figure S5: ^{1}H NMR (A) and ^{13}C NMR (B) spectra of compound **11** recorded in $CDCl_3$ at 300K

(A)

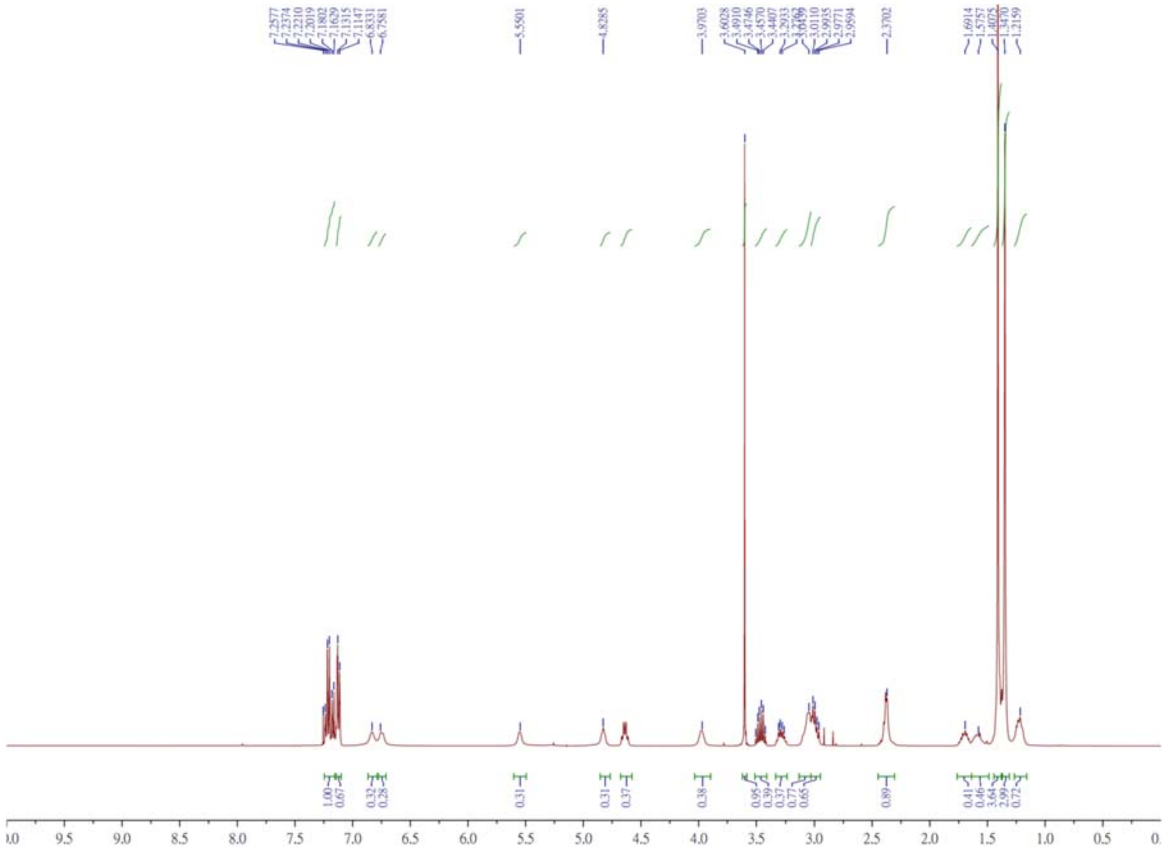

(B)

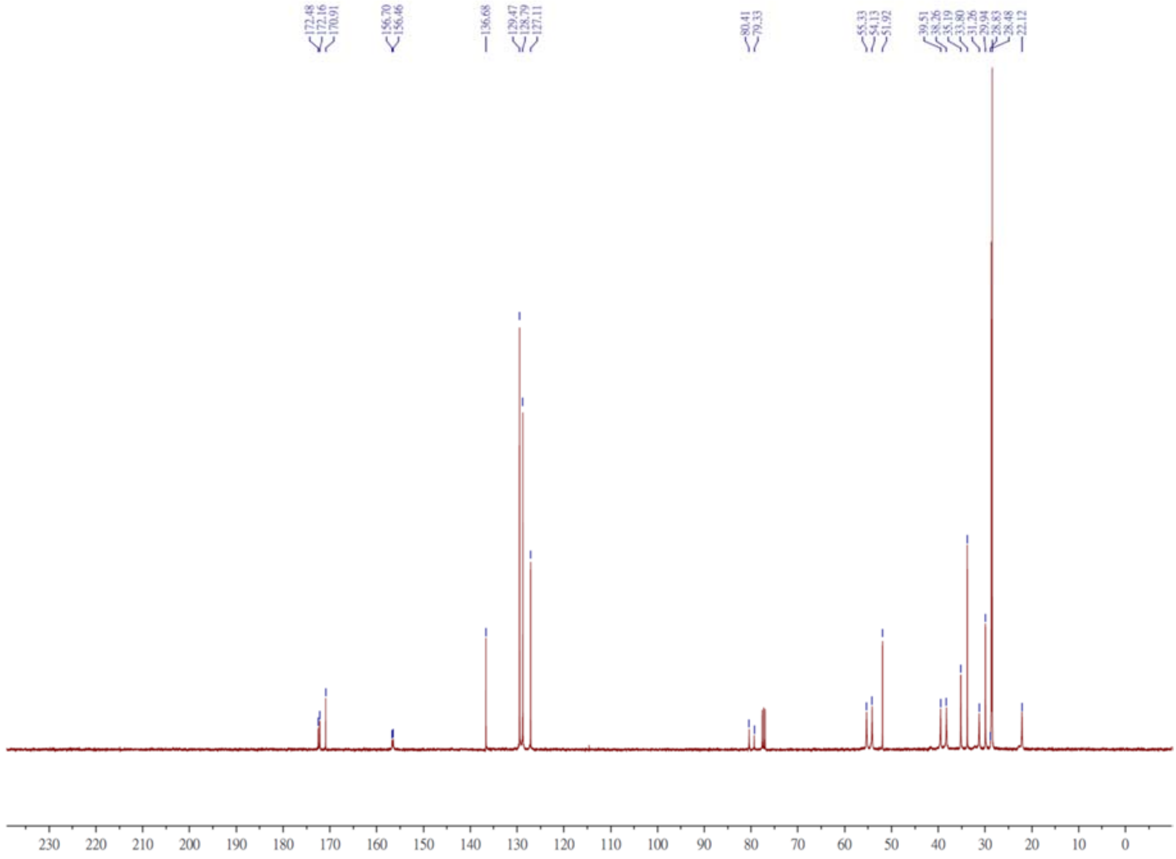

Figure S6: ^{1}H NMR (A) and ^{13}C NMR (B) spectra of compound **12** recorded in $CDCl_3$ at 300K

(A)

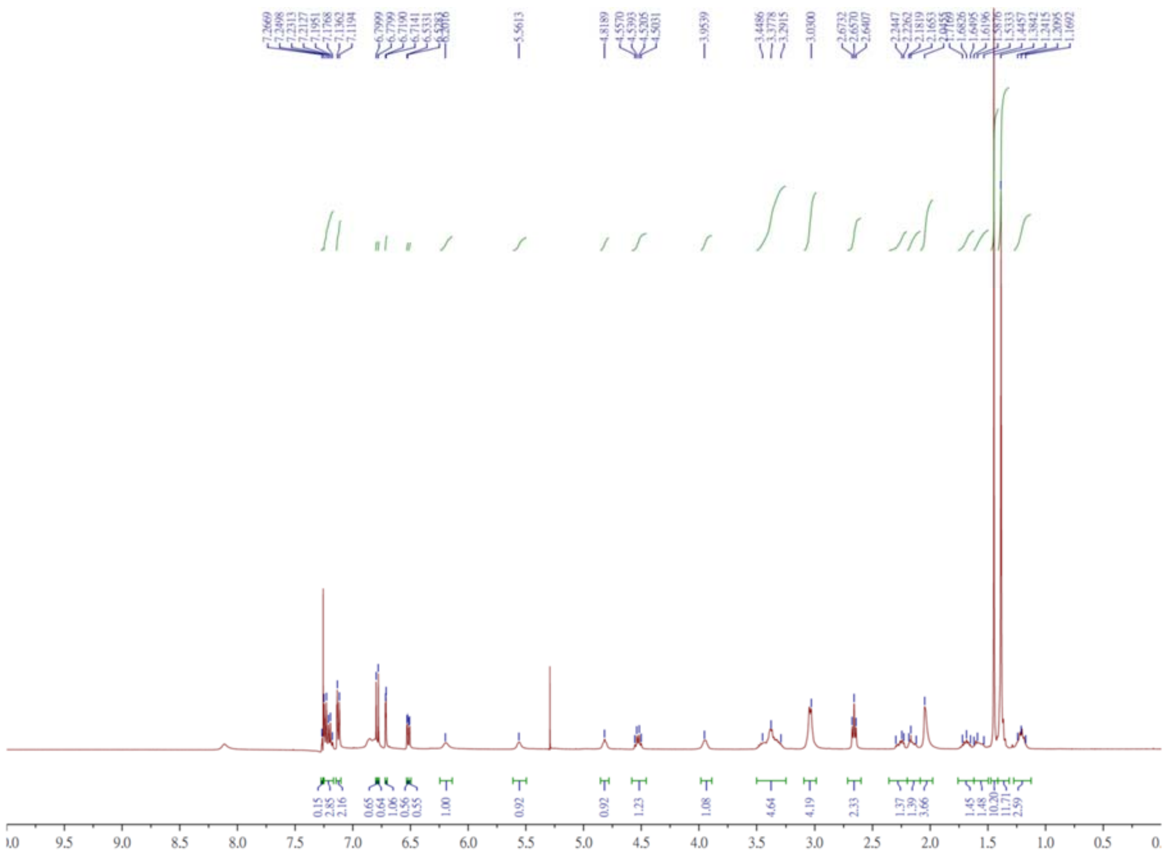

(B)

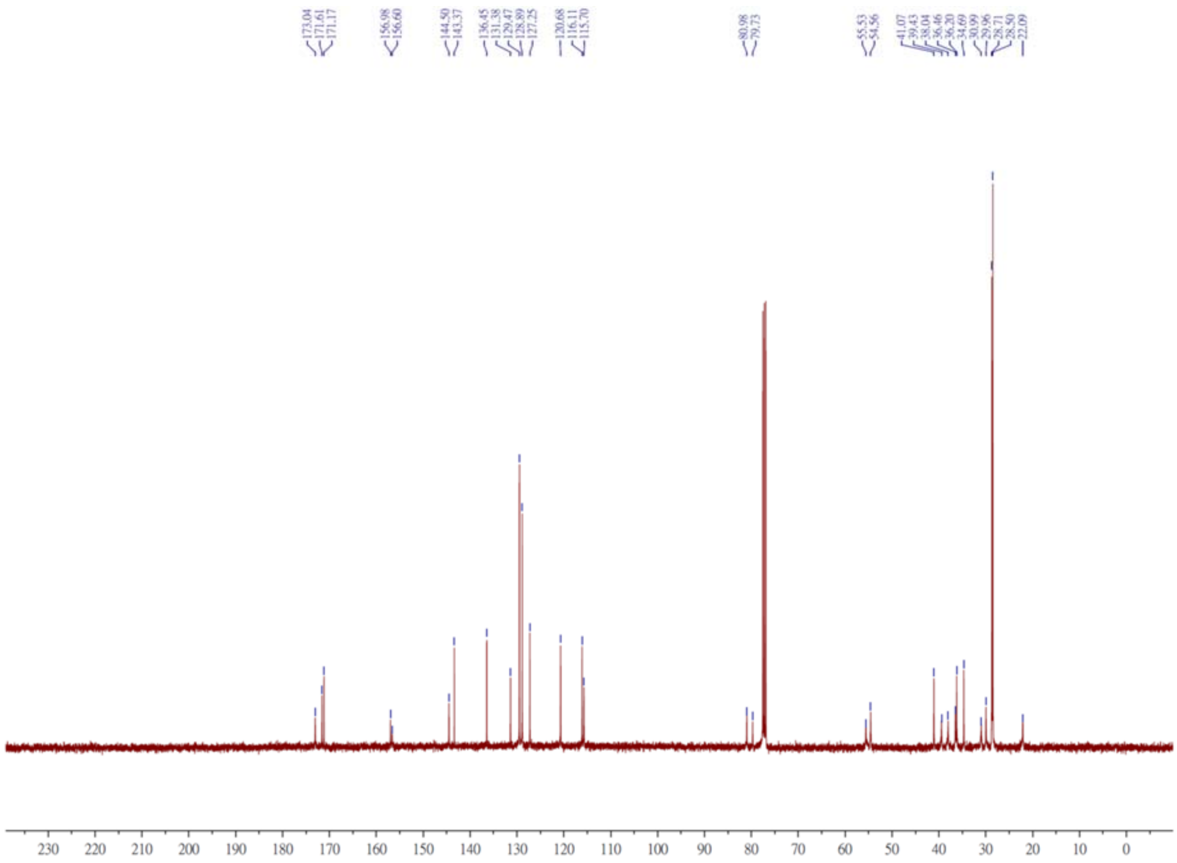

Figure S7: ^{1}H NMR (A) and ^{13}C NMR (B) spectra of compound **13** recorded in $CDCl_3$ at 300K

(A)

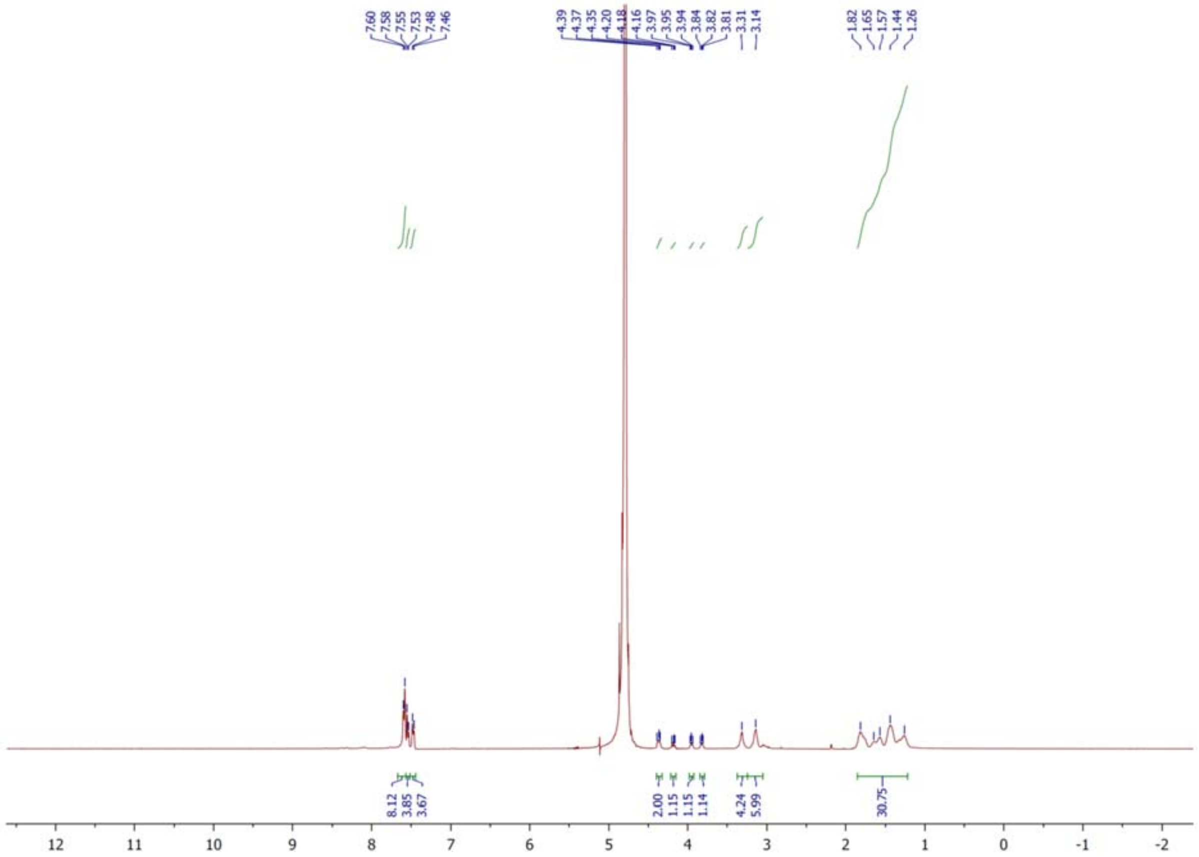

(B)

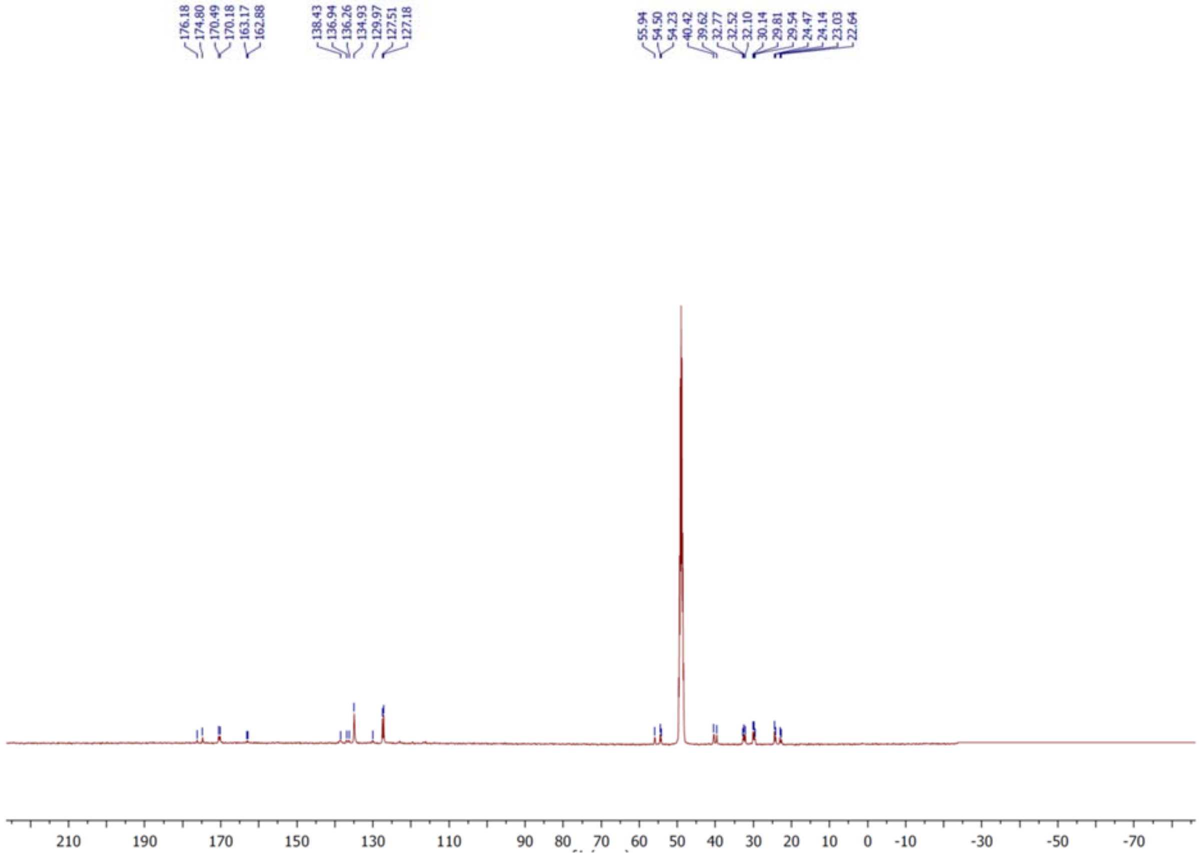

Figure S8: ^{1}H NMR (A) and ^{13}C NMR (B) spectra of compound **16** recorded in D_2O at 300K

III. ^{11}B NMR spectra and deconvolution results of dendrimers with TBAF

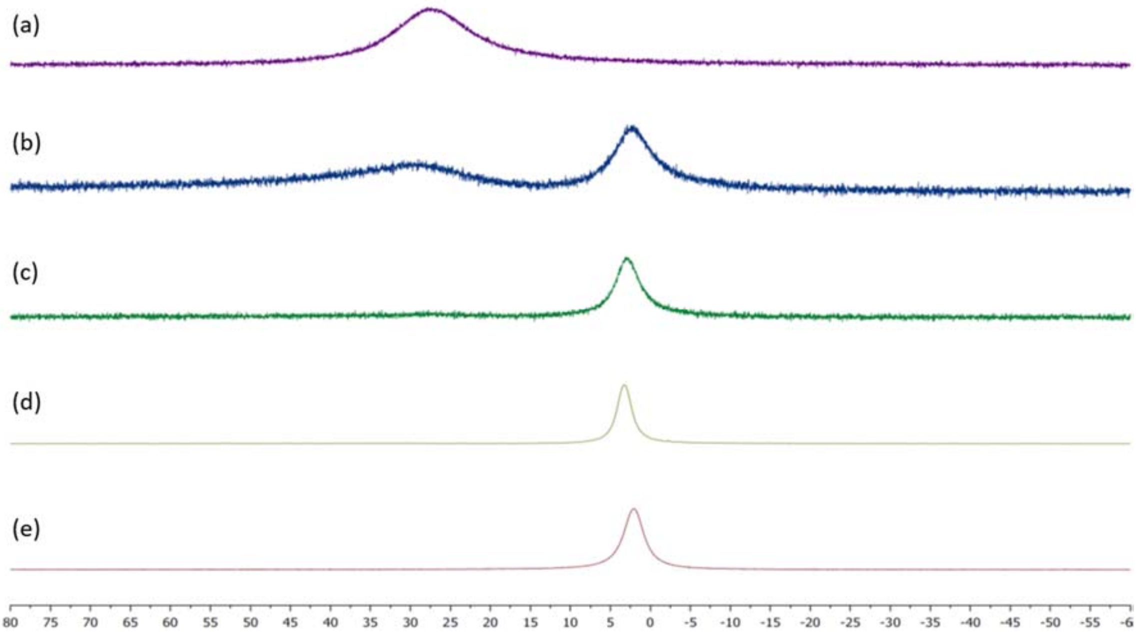

Figure S9: ^{11}B-NMR spectra of (a) boronic acid **1a**, (b) **1a** titrated with TBAF (1 equiv), (c) **1a** with TBAF (3 equiv), (d) tetra-*n*-butylammonium phenyl trifluoroborate (**3a**), (e) potassium (4-carboxyphenyl)trifluoroborate (**3b**). All spectra were acquired in DMSO-d_6.

(A)

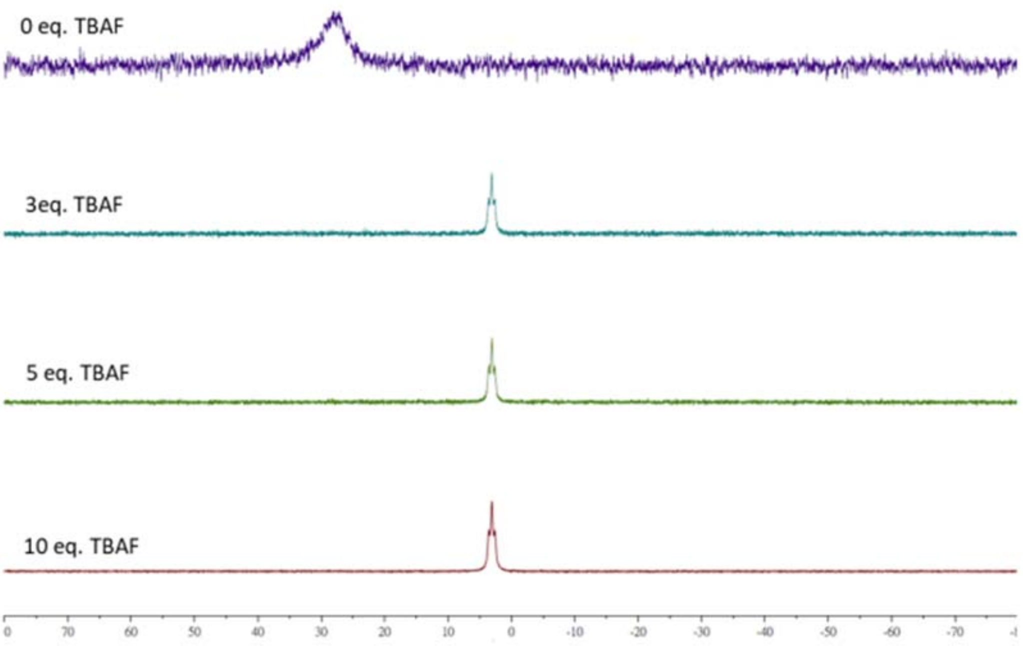

(B)

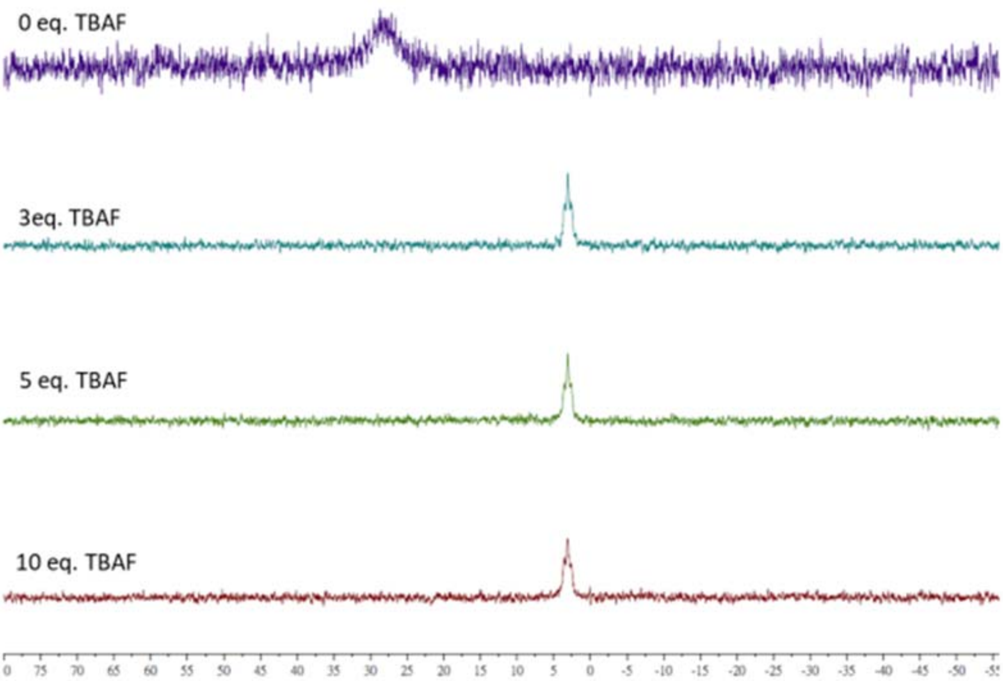

Figure S10. ^{11}B NMR spectra of 1 mM of ARS mixed with (a) 16 mM (b) 4 mM of PBA in 5 min incubation time with 3, 5, 10 equiv of TBAF. All spectra were acquired in DMSO-d_6.

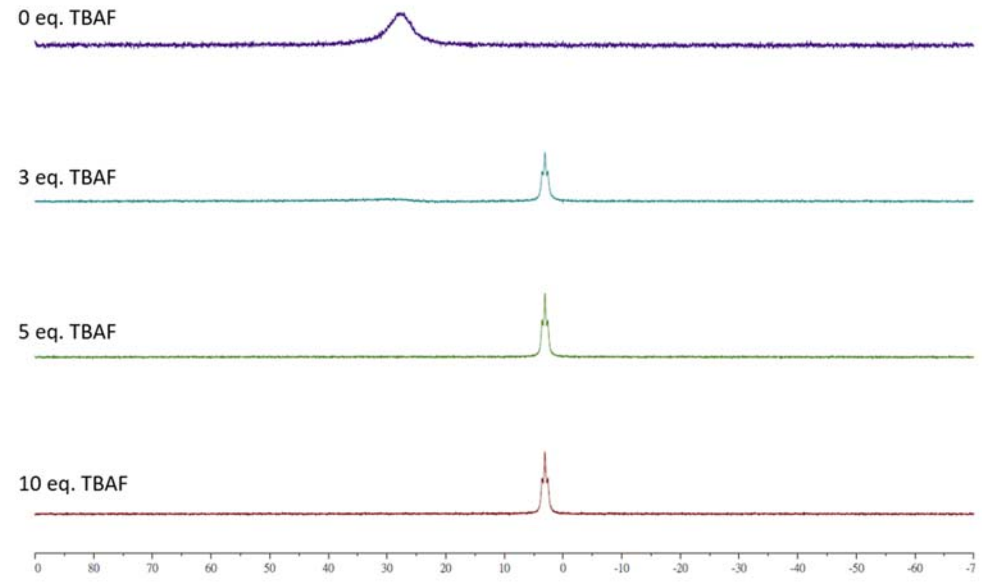

Figure S11. ^{11}B NMR spectra of 1 mM of ARS reacted with 4 mM of PBA in 30 min, 150W by microwave with 3, 5, 10 equiv of TBAF. All spectra were acquired in DMSO-d_6.

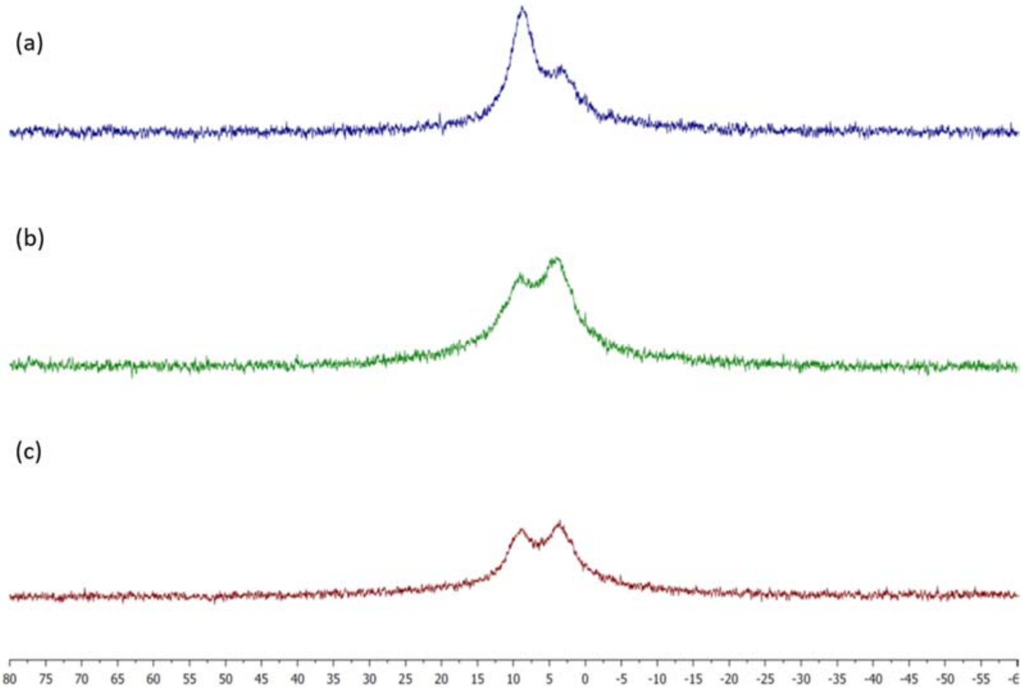

Figure S12: ^{11}B-NMR spectra of boronate ester **8a** with TBAF (3 equiv), (a) before purification, (b) after Sephadex® LH-20 column purification, (c) after precipitation by EtOH/ether. All spectra were acquired in DMSO-d_6.

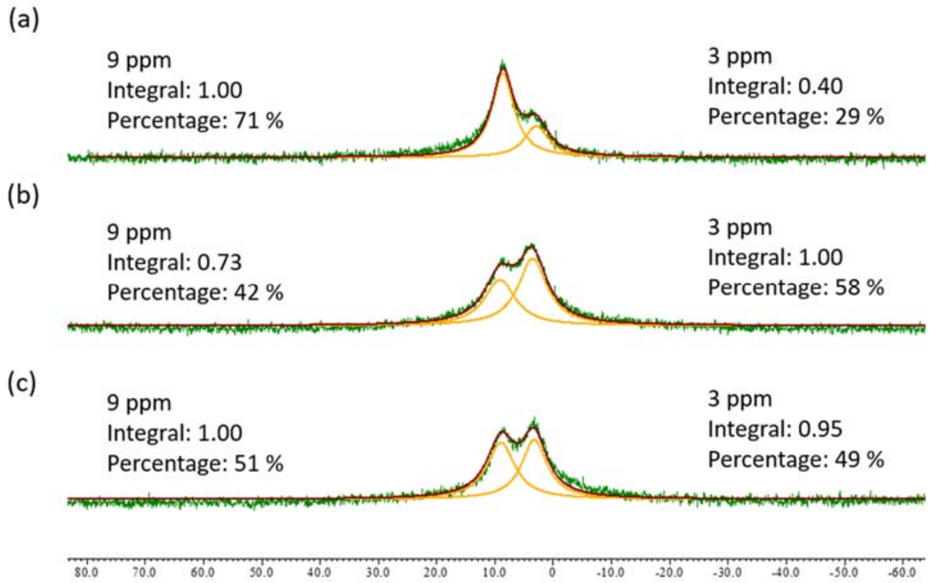

Figure S13: Deconvolution result for **Fig. S12**; ^{11}B-NMR spectra of boronate ester **8a** with TBAF (3 equiv), (a) before purification, (b) after Sephadex® LH-20 column purification, (c) after precipitation by EtOH/ether. All spectra were acquired in DMSO-d_6.

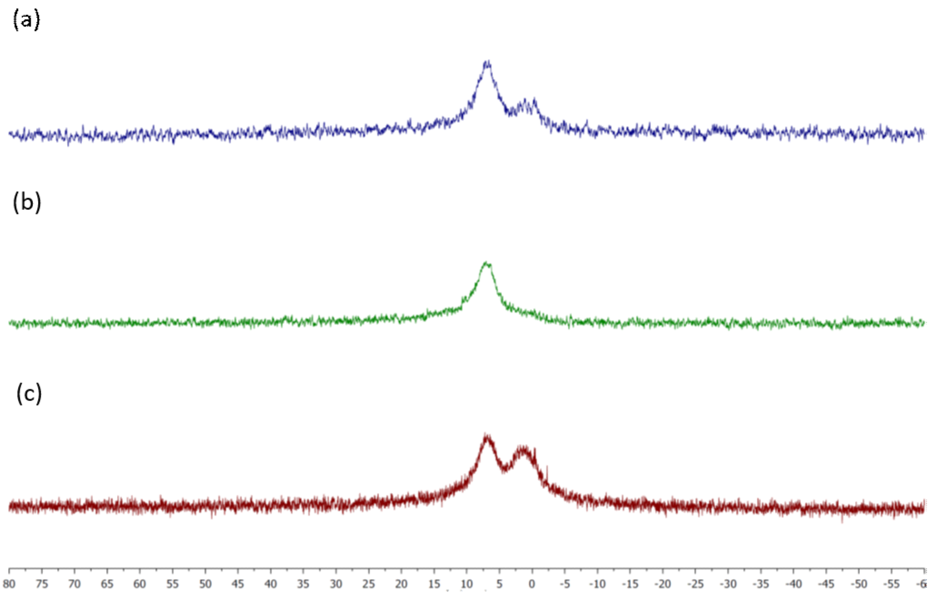

Figure S14: ^{11}B-NMR spectra of boronate ester **8** at various conditions of equiv of catechol **7** and reaction time. All the sample were added TBAF (3 equiv), (a) **7** to **6** (2:1), reaction time: 1 d (Table 2, entry 1), (b) equiv of **7** to **6** (1:1) (Table 2, entry 2), reaction time: 5 d, (c) equiv of **7** to **6** (2:1) (Table 2, entry 3), reaction time: 5 d. All spectra were acquired in DMSO-*d*$_6$.

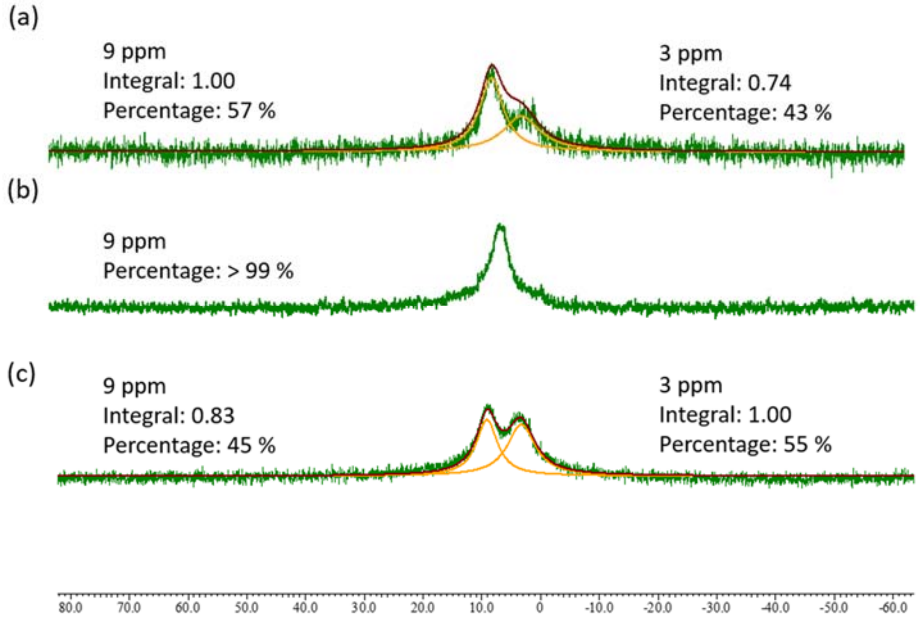

Figure S15: Deconvolution result for **Fig. S14**; ^{11}B-NMR spectra of boronate ester **8** with TBAF (3 equiv), (a) **7** to **6** (2:1), reaction time: 1 day (Table 2, entry 1), (b) equiv of **7** to **6** (1:1) (Table 2, entry 2), reaction time: 5 d, (c) equiv of **7** to **6** (2:1) (Table 2, entry 3), reaction time: 5 d. All spectra were acquired in DMSO-*d*$_6$.
